# The Mechanistic Roles of ncRNAs in Promoting and Supporting Chemoresistance of Colorectal Cancer

**DOI:** 10.3390/ncrna7020024

**Published:** 2021-03-31

**Authors:** Isaac Micallef, Byron Baron

**Affiliations:** Centre for Molecular Medicine and Biobanking, University of Malta, MSD2080 Msida, Malta; isaac.micallef.17@um.edu.mt

**Keywords:** ncRNAs, miRNAs, lncRNAs, circRNAs, colorectal cancer, chemoresistance, 5-fluorouracil, oxaliplatin, cisplatin, doxorubicin

## Abstract

Colorectal Cancer (CRC) is one of the most common gastrointestinal malignancies which has quite a high mortality rate. Despite the advances made in CRC treatment, effective therapy is still quite challenging, particularly due to resistance arising throughout the treatment regimen. Several studies have been carried out to identify CRC chemoresistance mechanisms, with research showing different signalling pathways, certain ATP binding cassette (ABC) transporters and epithelial mesenchymal transition (EMT), among others to be responsible for the failure of CRC chemotherapies. In the last decade, it has become increasingly evident that certain non-coding RNA (ncRNA) families are involved in chemoresistance. Research investigations have demonstrated that dysregulation of microRNAs (miRNAs), long non-coding RNAs (lncRNAs) and circular RNAs (circRNAs) contribute towards promoting resistance in CRC via different mechanisms. Considering the currently available data on this phenomenon, a better understanding of how these ncRNAs participate in chemoresistance can lead to suitable solutions to overcome this problem in CRC. This review will first focus on discussing the different mechanisms of CRC resistance identified so far. The focus will then shift onto the roles of miRNAs, lncRNAs and circRNAs in promoting 5-fluorouracil (5-FU), oxaliplatin (OXA), cisplatin and doxorubicin (DOX) resistance in CRC, specifically using ncRNAs which have been recently identified and validated under in vivo or in vitro conditions.

## 1. Introduction

Cancer is a disease which can develop anywhere in the body and arises due to changes occurring at a genetic level. Typical changes taking place in promoting cancer development include, mutagenesis, disruption of tumour suppressor genes and oncogene activations [1]. These malfunctions cause the cells to reproduce and grow aggressively, giving rise to a tumour. The third most common cancer in most Western countries is colorectal cancer (CRC), which is typically located in the rectum or colon of said individual [2]. This cancer starts off with an adenomatous polyp in the epithelial cells of the colon or rectum, which arises due to mutations in the tumour suppressor gene adenomatous polyposis coli (*APC*). Malignant features are not acquired by all polyps but the ones which develop additional alterations in other tumour suppressor genes and oncogenes can eventually become an adenocarcinoma, which may take several years or decades to develop [3]. CRC can be divided into three families; sporadic, hereditary and inflammation dependent CRC, each arising due to alterations in molecular mechanisms involved in cancer development [3,4,5]. Our knowledge on how this cancer develops has increased throughout the years, however mortality risk of CRC keeps increases [6].

The currently used CRC treatment approaches include surgery, chemotherapy, and radiotherapy simultaneously. The main scope of chemotherapy is to interfere with the cell’s metabolism, DNA replication, mitosis, and protein synthesis of the cancerous cells to hinder its growth [7]. Undoubtedly, one of the barriers commonly encountered throughout CRC treatment is the development of chemoresistance, which eventually leads to chemotherapy failure, metastasis, tumour recurrence and unfortunately patients’ death. Several chemotherapeutic drugs have been approved to interfere with tumour growth, with the main ones for CRC being 5-fluoruracil (5-FU) and its analogues, Oxaliplatin (OXA), Capecitabine and Irinotecan [8]. However, other modes of treatment have also been considered for CRC, such as Cisplatin and Doxorubicin (DOX) [9,10]. Most of these chemotherapeutic drugs function by interfering with the synthesis of RNA and DNA [11]. The antimetabolite pyrimidine analogue 5-FU [12] does so by incorporating itself into the DNA or RNA and by inhibiting the thymidylate synthases (TYMS) enzyme responsible for synthesising deoxythymidine monophosphate (dTMP) [13]. The platinum drugs, OXA and cisplatin interfere with the synthesis of DNA by acting as intercalating agents, forming intrastrand adducts between two purine residues (two guanine residues or between an adenine and guanine residue) [14,15]. With regard to DOX, its complex structure enables it to function via a number of mechanisms, interfering with DNA and RNA synthesis by intercalating itself between the strands, generating free radicals and reactive oxygen species (ROS), altering the cell membrane, and overproducing ceramide [16]. Despite these different chemo drugs being effective, chemoresistance has still shown to develop for each of the respective drug. Several studies have been conducted to identify the mechanisms of chemoresistance and in turn find solutions to overcome this fundamental problem. Chemoresistance in CRC has shown to generally arises due to different mechanisms, such as ATP-binding cassette (ABC) transporters, different signalling pathways (Wnt/β-catenin, Phosphatidylinositol-4,5-bisphosphate 3-kinase/Protein Kinase B (PI3K/AKT), Epithelial Growth Factor Receptor-Rat Sarcoma-Mitogen Activated Protein Kinase (EGFR-RAS-MAPK), Vascular Endothelial Growth Factor/Vascular Endothelial Growth Factor Receptor (VEGF/VEGFR) and Nuclear factor kappa-light-chain-enhancer of activated B cells (NF-κB)), apoptosis, autophagy, cell cycle control, specific drug targets, DNA damage repair, epithelial mesenchymal transition (EMT) and drug metabolism [12,17,18,19,20,21]. Despite the identification of all these mechanisms, the precise mechanisms causing CRC chemoresistance remain to be elucidated.

Throughout the past two decades or so, there have been an increasing number of studies reported on non-coding RNAs (ncRNAs) and their cellular functions. Studies have reported that disruption of ncRNA function via mutations, transcriptional and post-translational modifications, tend to be play a role promoting cancer development. In addition, alterations in ncRNA expression have shown to also be involved in regulating several protein targets or molecular pathways which eventually lead to or inhibit drug resistance [22]. Among the different ncRNAs identified to date, microRNAs (miRNAs) and long ncRNA (lncRNAs) are the two most studied ncRNAs involved in CRC chemoresistance, with circular RNAs (circRNAs) also gaining interest lately [20,23,24,25,26,27]. This review article will focus on highlighting and summarising some of the recently identified/validated key molecular players from three ncRNA families; mainly miRNAs, lncRNAs and circRNAs and the molecular mechanisms by which these ncRNAs affect CRC chemoresistance and sensitivity for the drugs 5-FU, OXA, Cisplatin and DOX. Along with this, a brief overview of the mechanisms of chemoresistance arising in CRC will also be discussed. The tumor-specific miRNAs, lncRNAs and circRNAs to be discussed may eventually serve as novel therapeutic targets and prognostic biomarkers for CRC in the future.

## 2. Overview of ncRNAs

**Figure 1:**
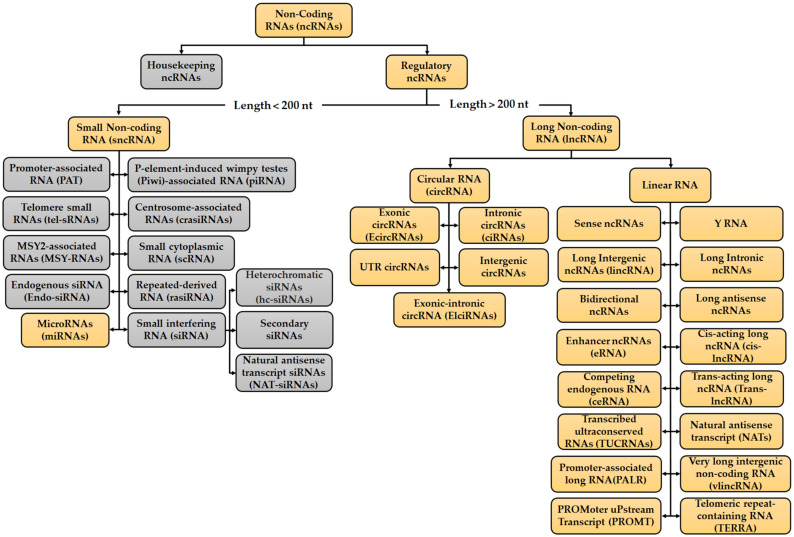
Classification of the Non-coding RNA (ncRNA) families. Two types of ncRNAs have been identified, Housekeeping ncRNAs and Regulatory ncRNAs. Despite the numerous types of ncRNAs identified, the focus in this review will be on the ones highlighted in orange as the ncRNAs to be discussed in the chemoresistance sections fall under these categories. Further work is to be done on the ncRNAs present in the grey boxes in order to see their involvement in CRC chemoresistance. Information for figure retrieved from: [31,38,40,41,47,48,49,50,51,52,53,54,55,56,57,58].

RNAs provide both catalytic and informative functions. With the rapid, increased development of high throughput methods and transcriptome sequencing techniques, scientists have gained a better understanding of both the protein-coding and non-coding portions of the mammalian transcriptome. Most of the mammalian genome is actively transcribed with approximately 2% of the genome encoding proteins [28,29,30]. The remainder of the human genome is known to harbour ncRNAs [31]. NcRNAs are involved in controlling gene expression before, after and throughout transcription, thus these RNAs participate in regulating and modulating different stages tumour progression, namely cell proliferation, migration, metastasis and chemoresistance [32]. The advances in the field of ncRNA have been reviewed from different mechanistic perspectives [33,34,35] and biological roles [30,36,37]. Eukaryotic transcription can generate different ncRNA species, which arise from different genomic regions and RNA processing [38]. Furthermore, different parts of the DNA can be transcribed into ncRNAs, for example protein-coding genes, transposon elements or enhancer regions [38,39]. Considering their average size and their regulatory role, ncRNA are split up into two groups: housekeeping ncRNAs and regulatory ncRNAs which are then further subdivided (Figure 1) [38,39,40,41]. Multiple kinds of ncRNAs have been identified and validated within these two groups, together with their vital functions in both pathological and physiological processes. Most of the identified ncRNA have been linked to different cellular functions, such as gene expression, proliferation, cell cycle progression, apoptosis, and various other functions [23]. Housekeeping ncRNAs are small (ranging from 50 to 500 nucleotides), constitutively expressed in all cell types and needed for cell viability [38,39]. Furthermore, this group primarily regulates generic cellular functions [39,40,41,42,43,44]. The regulatory ncRNAs can be further categorized into two groups based on their size: small non-coding RNAs (sncRNAs), which have transcripts not bigger than 200 nt and long noncoding RNA (lncRNAs) which have transcripts greater than 200 nt [38,39] (Figure 1). These act as key regulatory RNA molecules, which function as gene expression regulators at an epigenetic, transcriptional and post transcriptional level [38,42,43,44]. Even though ncRNAs are not present in great abundance and were referred to as ‘junk’ DNA, these still have important roles in transcription, post-transcriptional processing, and translation and are also constitutively expressed and essential for normal function of the cell [42,45,46].

The majority of the currently published/reported studies are on miRNAs and lncRNAs. However, in addition to miRNA and lncRNA, another type of ncRNA, circRNA, has also recently became one of the research focuses. MiRNAs are said to be the most abundant class of small ncRNAs, produced from transcribed hairpin loop structures [59,60]. MiRNAs contribute to regulating gene expression in the nucleus and cytoplasm via different mechanisms and mediate gene silencing at a post-transcriptional level in many human cellular contexts and diseases [38,59]. When carrying out their functions, miRNAs tend to also interact with other types of ncRNAs, mainly with lncRNAs and circRNAs, as these help regulate miRNA stability [59,60,61]. Furthermore, they contribute to differentiation and development (cell growth and survival) in human cells by completely or incompletely binding to the 3′-untranslated regions (3′-UTRs) of target messenger RNAs (mRNAs), which results in mRNA degradation or post-transcriptional inhibition [62]. In addition, miRNAs also contribute to regulating different biological pathways, including the cell cycle, apoptosis, differentiation, DNA repair, energy metabolism, proliferation, immune response, and stress tolerance [63,64]. LncRNAs are mostly transcribed by RNA polymerase II, but there are some cases in which lncRNAs are generated from the antisense region of upstream promoters [65,66]. They do not have an open reading frame, often harbor a poly-A tail, can be spliced, and are generally found in the cytoplasm and nucleus of a cell [46,65,67,68]. LncRNAs found in the nucleus are functionally involved in gene regulatory procedures, such as promoter specific repression or activation of transcription [34,69,70,71] or epigenetic gene regulation [72,73] and those found in the cytoplasm modulate post-transcriptional gene regulatory processes [7,37,46,74,75,76]. In addition, they modulate protein interactions, stability and affect their cellular localisation, together with controlling signalling cascades and any changes in gene expression [73]. CircRNAs are a category of endogenous ncRNA molecules which are widely expressed in eukaryotic cells and exhibit both location- and step-specificity [77,78]. As their name implies, they are circular (closed loops) and arise from splicing events and are one of the ncRNAs which can be generated from protein coding regions, but can also be synthesised from introns, intergenic regions, untranslated regions or from tRNAs [38,79,80]. This class of ncRNAs are mostly found in the cytoplasm, however a few can reside in the nucleus with most circRNAs having a conserved sequence [79,81]. Just like the other regulatory ncRNAs, circRNAs are involved in different biological processes; (1) interact with RNA binding proteins since circRNAs harbour sites for these proteins which can serve as protein sponges or decoys during gene expression [82], (2) bring about epigenetic alterations [83], (3) act as regulators for transcription or post-transcription and alternative RNA splicing [84,85], (4) translate proteins [78]. Despite their functional roles in cells, their biological roles remain largely speculative. Something common between both lncRNAs and circRNAs is that they can both act as miRNA sponges or competing endogenous RNAs (ceRNAs) to competitively bind to and sequester miRNAs, decreasing their regulatory effect on mRNAs [73,86,87,88]. CeRNAs refers to different ncRNAs (e.g., circRNAs and lncRNAs) which compete (through interactions/crosstalk) for the same pool of miRNAs, thus in turn regulate the activity of miRNAs [89]. miRNA sponge is another term used instead of ceRNAs and refers to those ncRNAs which attract miRNAs for binding and competitively sequester them from the miRNAs natural target/s due to having multiple tandem high-affinity binding sites to that miRNA of interest [90]. For both cases (miRNA sponges or ceRNAs), the miRNAs targeted will reduce its regulatory effect on mRNAs due to miRNAs being negatively regulated by other ncRNAs, thus releasing the inhibition of the mRNA being targeted by the miRNAs so degradation or translation can take place.

The normal function and expression of the different ncRNAs are vital for maintaining physiological conditions. However, if there is any disturbance in the function or abnormal expression of said ncRNAs, these can contribute to the development of pathological events, mainly cancer. Several studies have shown that aberrant expression of miRNAs, lncRNAs and circRNAs may participate in CRC progression via different mechanisms and in turn contribute to cancer cell growth and proliferation, apoptosis, metastasis, angiogenesis, and EMT transition [30,46,59,68,91,92,93,94,95,96,97,98,99,100,101,102,103,104,105,106,107,108,109,110,111,112,113]. This dysregulation results in the alteration of several signalling pathways, but due the complex structural characteristics demonstrated by ncRNAs, further structural, functional, and mechanistic characterisations are required to get a better understanding of their roles in cancer development [114]. Furthermore, throughout cancer development these three ncRNAs can act as oncogenes, proto-oncogenes or as tumour suppressors, with some ncRNAs also acting as both oncogenes and tumour suppressors [40,115,116,117,118,119,120,121,122]. Research shows that apart from ncRNAs being dysregulated, they can potentially be used as diagnostic and prognostic markers when collected from tissues, plasma, or serum for specific cancers such as CRC, pancreatic cancer, breast cancer, leukemia, lung cancer and various others [123,124]. Furthermore, due to having tissue specific signatures and due to the expression patterns, they demonstrate in tumours, ncRNAs have shown to be promising for the generation of accurate non-invasive biomarkers for prognosis and diagnosis [114]. Most of the ncRNA families have shown to contribute to not only cancer development but can also give rise to chemoresistance [23,26,125,126,127,128], and the coming sections will describe chemoresistance arising due to these three ncRNAs in relation to CRC.

## 3. Drug Resistance in CRC

Drug resistance is the decrease in effectiveness for drugs including chemotherapeutic agents, antibiotics, and antivirals, throughout the treatment of different diseases. A tumour can be intrinsically drug resistant from the start of treatment or can develop resistance throughout the course of said treatment [11,17]. Chemoresistance can be divided into two: single drug resistance and multidrug resistance (MDR). MDR refers to the development of resistance by cancer cells to anticancer drugs having different structures and functions [129]. This phenomenon typically arises due to various mechanisms and involves intrinsic and extrinsic factors, such as drug inactivation, EMT, DNA repair alterations and epigenetics, inhibition of cell death related pathways (apoptosis, senescence, necrosis, autophagy), drug efflux and reduced drug uptake, intracellular signalling pathways, changes in membrane lipids, tumour microenvironment, cancer stem cells (CSCs) and membrane transporters [130,131,132,133]. Different mechanisms have been shown to contribute to chemoresistance in CRC when patients are treated with the currently available CRC treatments, such as 5-FU and its analogues, OXA, Cisplatin or DOX. Given the accumulating literature regarding this field, the mechanisms which have shown to give rise to chemoresistance in CRC will be discussed below.

Transport based cellular mechanisms of drug resistance have been shown to be one of the most understood modes of resistance in CRC [12,17,18,20,21]. It mainly refers to the efflux of drugs out of cancer cells through different membrane transporters, thus resulting in decreased intracellular accumulation of anticancer drugs and chemotherapy failure. Numerous membrane transports which control the transport of different substrates into and out of the cell have been identified. The two major superfamilies are (1) ABC transporters, which are further divided into P-Glycoprotein (P-gp), breast cancer resistance protein (BCRP) and multidrug resistance-associated proteins (MRPs) and (2) solute carrier (SLC) transporters, which include organic anion transporters, organic cation transporters and organic anion transporting polypeptides [18,21]. To date, 48 ABC transporters have been identified in the human genome, which are further subdivided into seven families (A–G). ABC transporters are responsible for facilitating the efflux of excessive intracellular drugs, thus giving rise to a significant impairment of chemotherapeutic effects [134,135]. The ABC transporter families play major roles in chemoresistance arising in CRC, due to their overexpression [18,20,21]. For instance, overexpression of the ABCB1 efflux transporter gave rise to 5-FU resistance in CRC cell lines [136], while most of the ABC transporters are responsible for DOX resistance [16]. Furthermore, upregulation of MRP2 and BCRP was also involved in the cisplatin-induced drug resistance in CRC cell lines [137], while overexpression of MRP2 was reported to be responsible for cisplatin resistance in patients undergoing chemotherapy and for OXA resistance in CRC cell lines [138].

Non-transport-based mechanisms can also give rise to resistance in CRC and are often linked to altered activities of specific enzymes and alteration in different signalling pathways and cell death pathways. Apoptosis and autophagy are two programmed cell death pathways triggered by the cells. Apoptosis is initiated via different extracellular and intracellular signals while autophagy is initiated due to different stressful stimuli [18]. Evasion of apoptosis, one of the hallmarks of human cancers, contributes to carcinogenesis and tumor progression, as well as drug resistance in cancer. Resistance to apoptosis in cancer cells, in this cases CRC, is linked to increased expression of antiapoptotic genes and proteins, as well as decreased expression of pro-apoptotic genes and proteins [139]. Thus, Bcl-XL, Mcl-1, B-cell Lymphoma (Bcl-2) and X-linked inhibitor of apoptosis tend to be overexpressed in CRC, while Bax, p53, Bim and apoptotic protease activating factor 1 are mutated or suppressed [139]. This was in fact proven by different research groups, for example CRC cell lines were resistant to DOX and apoptosis due to the Bcl-2 protein level being significantly upregulated as compared to the parental cell lines while the expression of p53 and BAX were significantly downregulated [140]. Moreover, loss of Bax expression was shown to decrease the sensitivity of HCT116 cells to apoptosis induced by 5-FU and OXA [18]. The role of autophagy in tumorigenesis is still controversial. Autophagy can promote the survival of rapidly growing CRC cells by targeting damaged or aged organelles for degradation and recycling [18]. At later stages of carcinogenesis, autophagy may act to stimulate tumor expansion by providing energy and nutrients important to the metabolism and growth of malignant cells, or by increasing drug resistance [21]. The p38MAPK pathway plays a key role in autophagy, especially in cellular responses to 5-FU. Studies reported that the inhibition of this pathway relates with a decrease in 5-FU-mediated apoptosis, stimulating CRC cell resistance. 5-FU resistance mediated by p38MAPK pathway inhibition is linked to autophagic response as it induces a decrease in p53-driven apoptosis without effect on p53-dependent autophagy. Consequently, the p38MAPK signaling pathway plays a critical role in CRC cell 5-FU resistance by controlling the balance between apoptosis and autophagy [141].

Apart from the cell death pathways, chemoresistance in CRC is also related to signaling pathways common to many other cell events. Thus far, several studies have indicated the relationship between different signaling pathways with chemoresistance of CRC cells. The Epithelial Growth Factor Receptor- Rat Sarcoma- Mitogen Activated Protein Kinase (EGFR-RAS-MAPK), Vascular Endothelial Growth Factor/Vascular Endothelial Growth Factor Receptor (VEGF/VEGFR), Phosphatidylinositol-4,5-bisphosphate 3-kinase/Protein Kinase B (PI3K/AKT), WNT/β-catenin, Nuclear factor kappa-light-chain-enhancer of activated B cells (NF-κB), and Notch1 signaling pathways seem to be the most important signaling pathways involved in chemoresistance of CRC cells [19,20]. The most common genetic changes associated with CRC progression are mutations that deregulate the Wnt/β-catenin signaling cascade. This pathway is essential for maintaining cell homeostasis and embryonic development, and is associated with tumor cell proliferation, apoptosis, invasion, stemness and chemotherapy resistance [142]. The AKT/PI3K signaling pathway is activated uncontrollably in CRC due to mutations in various components of this pathway, as well as mutations in inhibitors such as PTEN, which enhance chemoresistance [17,20,143,144]. The function of PTEN is to regulate the activity of PIP3 by dephosphorylation, and thus inhibit Akt activity. PTEN mutation and loss of its activity thus leads to constitutive activation of Akt, promotion of cell survival and enhances chemoresistance [17]. The VEGF/VEFGR pathway is related to angiogenesis and lymphangiogensis in tumour growth. The VEGF family consists of five members (VEGF-A, -B, -C, and -D and placental growth factor (PIGF)), which can bind to endothelial cells via tyrosine kinase VEGF receptors. Vascular endothelial growth factor receptors (VEGFRs) are split into three, VEGFR-1, -2, and -3, along with the non-tyrosine kinase coreceptors neuropilin-1 (NP-1) and NP-2 [19]. The VEGF family members can interact with different proteins to regulate angiogenesis. Over-expression of the VEGF gene and high levels of circulating VEGF protein are both associated with worse prognosis in CRC [17,19]. NF-κB is a ubiquitous transcription factor that mediates a cytoplasmic/nuclear signaling pathway and regulates gene expression of various cytokines, cytokine receptors and adhesion molecules involved in inflammatory and immune reactions. In addition, there are links between the initiation of NF-κB and control of apoptosis, proliferation, differentiation, migration, angiogenesis, and resistance to chemo and radiotherapies in CRC [145]. The Notch signalling pathway is involved in the maintenance and homeostasis of intestinal epithelium and controls the cellular fate of intestinal stem cells and differentiation of colonic goblet cells. In CRC, this pathway is critical for maintaining the balance between cell proliferation, apoptosis, and differentiation, and thus its dysregulation results in further progression of CRC [146]. In fact, dysregulation of the Notch signaling pathway contributed to CRC progression, metastasis, and inhibition of apoptosis [147]. Lastly, the EGFR-RAS-MAPK pathway participates in many cellular processes, including the growth, proliferation, and survival of normal cells. If dysregulated, it modulates the growth, survival, proliferation, metastasis and chemoresistance of neoplastic colorectal cells [144].

Some of these signalling pathways have been shown to display a relationship between the expression of ABC transporters, particularly the P-gp transporter and certain pathways. For instance, there is a relationship between P-gp and the NF-κB signaling pathway, and it seems that inhibiting this signaling pathway leads to P-gp downregulation [148]. In addition, the AKT/PI3K signaling pathway and P-gp upregulation have been shown to contribute to 5-FU resistance in CRC cell lines [149]. The Wnt/β-catenin signaling cascade also contributed to enhanced resistance to various chemotherapeutic agents in CRC through upregulating MDR1 as reviewed by Yuan et al. [142]. Apart from interacting with ABC transporters, the signalling pathways can crosstalk between themselves. For example, a study has shown the relationship between the Notch signaling pathway and 5-FU and OXA resistance in CRC cell lines [150]. The Notch signaling pathway exerts its effect on chemoresistance by interacting with the WNT/B-catenin signaling pathway [150]. Downstream target genes in the Wnt/β-catenin signaling cascade have been shown to regulate drug resistance by controlling apoptosis. For instance, MMP-7 (matrilysin, a metalloproteinase with prometastatic function) could increase the OXA resistance of colon cancer cells by decreasing the Fas receptor that stimulates cell apoptosis [142,151]. Activation of VEGFR-1 under pathological conditions mediates the activation of several downstream pathways, such as PI3K/AKT/MAPK/ERK, while the VEGFR-2 interacts with VEGF-A [19]. Upon activation of VEGFR-2, tyrosine residues are phosphorylated together with the initiation of different pathways, such as RAS/RAF/ERK/MAPK pathways, by which epithelial cell growth is promoted, and the PI3K/AKT pathway, by which cell apoptosis may be avoided and chemoresistance is promoted [19].

EMT, a process that drives a cellular trans-differentiation continuum under physiological conditions and pathological states, has also been shown to be one of the main reasons why cancers become resistant to the treatment being administered. During EMT, epithelial cells progressively lose their typical morphological features (cell polarity, cell-to-cell contacts, membrane adhesion) and develop a mesenchymal phenotype, with the typical cellular stellate morphology, different propensity for intercellular signaling, as well as overall distinct cytological and tissue architecture [21]. EMT in CRC malignant colonocytes may be induced by different stimuli arising from external sources such as transforming growth factor β (TGF-β), plus various cytokines working together with intracellular operative signalling cascades including PI3K and NF-κB, along with other stimuli [21,152]. However, EMT can also be induced by different cytotoxic drugs which are typically used for CRC. For example, CRC cell lines treated with DOX induced EMT cell phenotypes, TGF-β signaling, and a significant increase in P-gp expression, which gave rise to resistance [16,153]. Similarly, 5-FU-resistant CRC cell lines showed an increase in different mesenchymal markers, together with an increase in EMT-inducing transcription factors Zeb2, Zeb1 and Twist [154].

As previously mentioned, most of the chemotherapeutic drugs being discussed in this review function by interfering with the synthesis of RNA and DNA. Thus, DNA damage repair mechanisms can become an important contributor to drug resistance. The mismatch repair system (MMR) is a replication fidelity complex which is responsible for detecting and repairing any faults or mismatched bases arising in the cell throughout DNA replication and various other processes [155]. In CRC, mismatch repair deficiency (dMMR) can occur in both sporadic and hereditary CRC [12]. Germline mutations in either MutL homolog 1 (MLH1), MutS homolog 2 (MLH2), PMS1 Homolog 2 (PMS2), or mutS homolog 6 (MSH6) give rise to dMMR [12,156]. Epigenetic hypermethylation of the MLH_1_ promoter can also give rise to dMMR [155,157]. Different studies have been carried out to understand the relationship between MMR and the response of different CRC therapeutic agents in the development of resistance. It has been shown that cells which have dMMR tend to be resistant to certain cytotoxic agents which function by targeting the DNA. For instance, DNA mismatches produced by incorporation of 5FdUTP into DNA were not detected in dMMR cells, which result in cell survival and 5-FU resistance, while chemoresistance was reversed once MMR deficiency was corrected [156,158]. This phenomenon was also observed when using cisplatin in the study carried out by Martin et al. [159], while loss of either MSH2 or MLH1 function resulted in resistance to DOX [160]. Despite OXA being a platinum compound like cisplatin, OXA resistance in dMMR CRC cells lacking MMR is highly unlikely, as studies have shown that cisplatin–DNA adducts can be recognized and repaired by the mismatch repair system (MMR), whereas OXA–DNA adducts are not [161,162]. Thus, cells which have dMMR are intrinsically resistant to cisplatin but remain sensitive to OXA. However, OXA resistance in CRC can arise due the excision repair cross-complementing group 1 (ERCC1), and its catalytic partner Xeroderma Pygmentosum group F (XPF), which are key elements for the nucleotide excision repair (NER), responsible for repairing DNA adducts arising due to platinum compounds [14,163].

Lastly, based on the drugs being administered, chemoresistance in CRC can also arise due to drug metabolism and specific targets/enzymes [12,13,17]. These two modes of resistance tend to be specific to the drug being administered, since different chemotherapeutics are targeted or metabolised by different targets/enzymes. For example, 5-FU resistance tends to also arise due to malignant cells overexpressing dihydropyrimidine dehydrogenase (DPD), which is the enzyme responsible for the first step of 5-FU catabolism [13,17]. In fact, high levels of DPD mRNA expression in CRC cells have been associated with 5-FU intrinsic resistance [17,164,165]. On the other hand, if a low expression of DPD is present, 5-FU cannot be metabolised efficiently [12].

### 3.1. ncRNAs in Chemotherapeutic Resistance

**Figure 2:**
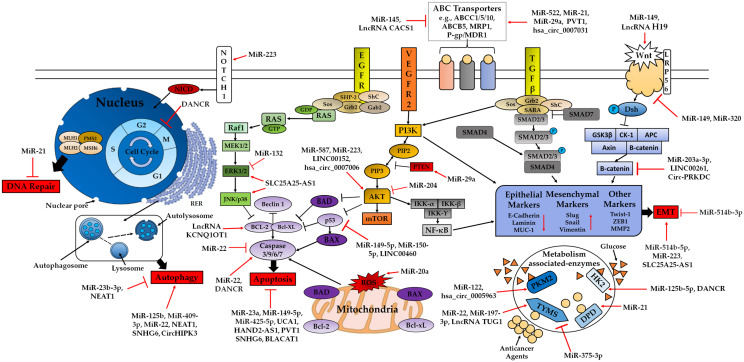
Classic mechanisms involved in drug resistance (5-FU, OXA, Cisplatin and DOX) in CRC. miRNAs, lncRNAs and circRNAs modulate drug resistance in CRC through different pathways such as the PI3K/AKT, EGFR-RAS-MAPK, WNT/β-catenin, NF-κB, TGF-β and Notch1 signaling pathways, cell death pathways (apoptosis, autophagy), EMT, DNA repair and ABC transporters. Arrows represent activation effect, and the ‘T’ symbols represent inhibition. Arrows and ‘T’ symbols in red represent some of the different ncRNA targets involved in chemoresistance to be discussed in this review. Abbreviations: MLH1: MutL homolog 1; MLH2: MutS homolog 2; PMS2: PMS1 Homolog 2; MSH6: MutS homolog 6; NICD: Notch intracellular domain; RER: Rough Endoplasmic Reticulum; GRB2: Growth Factor Receptor-bound protein 2; SOS: Son of Sevenless; *SHP*-*2*: SH2 domain-containing protein tyrosine phosphatase-2; GAB2: GRB2 Associated Binding Protein 2; SHC: SH2 containing protein; GSK3β: Glycogen Synthase Kinase 3β; AKT: Protein Kinase B; mTOR: Mammalian target of rapamycin; Iκκ: IκB kinase; Dsh: Dishevelled; PIP2: Phosphatidylinositol 4,5-bisphosphate; PIP3: Phosphatidylinositol (3,4,5)-trisphosphate; APC: Adenomatous Polyposis Coli; HK2: Hexokinase 2; TYMS: thymidylate synthases; DPD: Dihydropyrimidine Dehydrogenas; PKM2: Pyruvate Kinase 2; SARA: SMAD anchor for Receptor Activation.

Interestingly, over the years, ncRNAs have gained interest in this field due to also having pivotal roles in regulating resistance to different cancer treatments. ncRNAs are accountable for chemoresistance (Figure 2) as they play a part in (1) moderating drug resistance related genes, (2) affecting intracellular drug concentration, (3) initiating alternative signalling pathway, (4) promoting EMT, (5) altering drug efficiency by blocking DNA damage response, (6) preventing therapeutic-induced cell death and (7) altering cell cycle [22,166,167]. In general, ncRNAs contribute to chemoresistance in different cancers [168], such as gastric cancers [169,170], pancreatic cancers [171,172], breast cancers [173,174], glioblastomas [175,176], hepatocellular carcinomas [177,178], lung cancers [179,180], leukaemias [181,182] and ovarian cancers [183,184], among others. Despite all these cancers, the following sections will focus on how ncRNAs are involved in chemoresistance arising in CRC.

### 3.2. Drug Resistance in CRC Due to miRNAs, lncRNAs and circRNAs

The discovery of miRNAs, lncRNAs and circRNAs implicated in the response to antitumor therapy has been shown to promote the generation of new therapeutic approaches to reverse drug resistance. Despite our current understanding of the role of these ncRNAs in normal cells and in CRC development, as discussed previously, there are still issues to resolve, with one of them being the mechanism of action and role of these ncRNAs in the development of chemoresistance [27,166,185]. From the known ncRNAs (Figure 1), miRNAs and lncRNAs are the two most studied and understood ncRNAs involved in chemoresistance [23,24,26,27,70,116,186,187]. CircRNAs amongst the other ncRNAs have also been shown to be involved in chemoresistance [25,188,189,190], but how circRNAs contribute to chemoresistance in CRC is an area which is limited and requires more research when compared to the contribution of miRNAs and lncRNAs to CRC chemoresistance. The link between the activity of these three ncRNAs and the development of CRC chemoresistance has been studied and characterised by different research groups. Aberrant expressions for each of the aforementioned ncRNAs have indicated their involvement in the different mechanisms of chemoresistance discussed in the previous sections. Thus, it is crucial to delve deeper and understand the mechanism of action for the respective ncRNA in regulating 5-FU, OXA, cisplatin and DOX resistance via the regulation of signalling pathways and biological processes (Figure 2). The coming sections will provide a comprehensive overview of 5-FU, OXA, Cisplatin and DOX resistance arising in CRC due to these three types of ncRNA. The majority of the studies to be mentioned and discussed in this review are very recent ones and have thus been only carried out by one research group. Furthermore, most have been validated under both in vitro and in vivo conditions, although only by the respective research group performing the investigation, and thus will eventually have to be further investigated and validated. Besides the ones to be discussed here, there are various other miRNAs, lncRNAs and circRNAs which have been identified and reviewed in the literature and have also contributed to modulating sensitivity and chemoresistance in CRC for the aforementioned drugs [13,20,22,23,25,32,70,125,142,147,189,191,192,193].

#### 3.2.1. miRNAs, lncRNAs, circRNAs in 5-FU Resistance

As explained in the previous sections, 5-FU resistance in CRC has been a problem researchers have faced for the past two decades or so. Resistance due to this drug tends to arise mostly due to different members of the multidrug transporters’ family, EMT, specific targets such as TYMS and DPD, cell death related pathways (apoptosis, autophagy), cell cycle and several different signalling pathways (MAPK/ERK, PI3K/AKT, Wnt/B-catenin, Hippo, NF-κB, Notch) [17,147,194]. Based on the research carried out to date, the three ncRNAs being discussed here have been shown to play a part and contribute to 5-FU resistance via most of these mechanisms (Figure 2) as will be discussed below (Table 1).

##### ABC Transporters Family

One recent study which investigated the role of miRNAs on regulating ABC transporters in promoting 5-FU resistance, is that carried out by Wu et al. [195]. MiR-21 was shown to be upregulated in 5-FU CRC cell lines, while silencing of this miRNA decreased the IC50 of resistant 5-FU CRC cells and increased apoptosis. Their data shows that miR-21 can regulate and inhibit Programmed cell death protein 4 (PDCD4), while high expression of the PDCD4 protein negatively regulated the expression of the ABC transporter ABCC5 and the stem cell marker Cluster of Differentiation 44 (CD44). It was then concluded that miR-21 regulated 5-FU resistance by inhibiting the PDCD4, which in turn resulted in increased expression of ABCC5 and CD44 [195]. Furthermore, a more recent study confirmed that miR-21 downregulates PDC4 in CRC cells which are resistant to 5-FU [196]. Another research investigation carried out but Zhang et al. [197] demonstrates that 5-FU sensitivity can be increased if miR-361 is overexpressed. This miRNA was found to be decreased in 5-FU resistant CRC cell lines, while its overexpression sensitised the resist CRC cells to 5-FU and in turn induced apoptosis. The phenomenon was proven to arise due to miR-361 acting as a negative regulator of forkhead box M1 (FOXM1). In fact, FOXM1 knockdown increased caspase 3/7 activity while, its upregulation decreased the expression of said caspases. Furthermore, they also investigated whether inhibition of FOXM1 could affect the expression of ABCC10 and ABCC5 [197]. This is because a previous study had shown that FOXM1 evoked 5-FU resistance in CRC due to upregulating ABCC10 [198]. Zhang et al. [197] also demonstrated that decreased FOXM1 expression resulted in decreased expression of ABCC5 and ABCC10. In addition, CRC cells expressing upregulated miR-361 also exhibited a reduced expression of ABCC5 and ABCC10. Collectively, it was proven that the miR-361/FOXM1-ABCC5/10 axis could regulate sensitivity of 5-FU in CRC cells, with the data collected providing novel targets for the treatment of CRC patients which are resistant to 5-FU [197].

The lncRNA Plasmacytoma Variant Translocation (PVT1) was associated with 5-FU resistance in human CRC tissues and cells, through inhibition of apoptotic cell death and upregulation of Multidrug Resistance Protein 1 (MRP1), P-gp, mTOR, and apoptosis regulator Bcl-2 [199]. PVT1 was upregulated in 5-FU resistant CRC cells and tissues, while silencing of this lncRNA resulted in increased 5-FU sensitivity, as well as higher apoptotic cell death and lower rates of cancer cell survival. This study was the first to investigate the role of PVT1 in promoting MDR in CRC cells treated with 5-FU [199]. In the study by He et al., circ_0007031 upregulation was linked to 5-FU resistance in CRC and upon silencing circ_0007031, CRC cell proliferation was suppressed, and the cells were more sensitive to 5-FU [200], which was similar to what Xiong et al. [201] had previously reported. However, in this study, the data collected showed that resistance arose due to circ_0007031 modulating ABCC5 expression by directly interacting and acting as an miR-133b sponge. Thus, circ_0007031 gives rise to resistance by modulating the miR-133b/ABCC5 axis [200].

##### PI3K/AKT Signalling Pathway

Zhang et al. reported that miR-587 conferred resistance to 5-FU-induced apoptosis in vitro and reduced the potency of 5-FU in the inhibition of tumour growth in a mouse xenograft model [202]. This resistance arose due to miR-587 downregulating the expression of Protein Phosphatase 2 Scaffold Subunit A beta (PPP2R1B), a regulatory subunit of the PP2A complex, which negatively regulates phosphorylation of AKT. Furthermore, downregulation of PPP2R1B resulted in increased AKT phosphorylation, which was followed by an increased X-linked Inhibitor of Apoptosis (XIAP) expression and enhanced 5-FU resistance. This finding showed that miR-587/PPP2R1B/pAKT/XIAP signalling axis has an important role in mediating the response to 5-FU in CRC under both in vivo and in vitro conditions [202]. In another study, overexpression of miR-204 increased the sensitivity of CRC cells to 5-FU by targeting and suppressing the high mobility group protein A2 (HMGA2) [203]. Originally, miR-204 was reported to be downregulated in both CRC tissues and 5-FU resistant cells, while HMGA2 was upregulated. Furthermore, the study also proved that miR-204 could target HMGA2 by directly binding to its 3′ untranslated region. Additionally, when miR-204 was upregulated, the protein expression of p-PI3K and p-AKT was inhibited, while increased HMGA2 expression restored the effect of miR-204. It was thus proven that miR-204 could suppress the activation of the PI3K/AKT signaling pathway through HMGA2 in 5-FU treated cells. In conclusion, this study demonstrated that miR-204/HMGA2 could regulate the sensitivity of 5-FU partly through the PI3K/AKT signaling pathway [203].

The AKT signaling pathway was reported to be associated with 5-FU resistance in CRC cell lines [201]. The study by Xiong et al. showed that hsa_circ_0007031, hsa_circ_0007006 and hsa_circ_0000504 were upregulated in such resistant cells [201]. Such a phenomenon was proposed to have arisen due to these three upregulated circRNAs interacting and modulating AKT3 and its regulatory miRNAs, thus AKT signaling may be stabilized by such circRNAs. Despite this discover, more work has to be done to further confirm this speculation, as this was only based on bioinformatic analysis (using KEGG pathways) carried out [201].

##### MAPK/ERK Signalling Pathway and EMT

In the study by Li et al., a significant decrease in lncRNA SLC25A25 Antisense RNA 1 (SLC25A25-AS1) expression level was observed in both tissues and sera of CRC patients [204]. SLC25A25-AS1 downregulation was found to dramatically potentiate CRC proliferation, EMT and 5-FU resistance by activating the MAPK/ERK signalling pathway through Erk and p38. However, when SLC25A25-AS1 was overexpressed, it enhanced the sensitivity of 5-FU due to increased cytotoxicity. Furthermore, this lncRNA was proven to be associated with promoting EMT. In fact, CRC cell lines having downregulated SLC25A25-AS1 exhibited less epithelial features, while those having an overexpression of this lncRNA occurred with decreased mesenchymal characteristics. This phenomenon was said to have arisen through the Erk/MAPK pathway, since phosphorylation of Erk and p38 was elevated remarkably in cells having knocked down SLC25A25-AS1, while phosphorylation decreased when SLC25A25-AS1 was overexpression. It was established that SLC25A25-AS1 expression in CRC can influence 5-FU chemoresistance by controlling EMT changes through the Erk/MAPK signalling pathway, in both CRC tissues and cells, but further studies are to be performed as this is the only reported study performed on CRC [204].

##### DNA Repair Pathways

MiR-21 can drastically hinder G2/M cycle arrest and apoptosis stimulated by 5-FU [205]. This miRNA was proven to target proteins in the mismatch repair system, particularly hMSH2 and hMSH6, due to the authors identifying putative binding sites on miR-21. In turn, the protein expression for both hMSH2 and hMSH6 was downregulated in cells having overexpressed miR-21. The expression of the miRNA was upregulated in CRC tissues while that of hMSH2 was downregulated. No data was provided for the expression of hMSH6 as this was not further investigated. Furthermore, miR-21 overexpressed and cells having mutated *hMSH2* showed decreased sub-G1 (apoptosis) and G2/M cells upon treatment with 5-FU. Furthermore, 5-FU resistance in xenograft models was induced due to overexpression of the miRNA and downregulation of hMSH2. As a whole this indicated that downregulation of hMSH2 expression by miR-21 can give rise to 5-FU resistance and it suggested that miR-21 dependent downregulation of hMSH2-hMSH6 might be responsible for both primary and acquired resistance to 5-FU [205]. In fact, another research group [206] also observed similar results when investigating the role of miR-21 and hMSH2 in 5-FU resistance under in vitro conditions. Apart from verifying the results obtained by Valeri et al. [205], Deng et al. were also the first to report that miR-21 can indirectly interact and downregulate thymidine phosphorylase (TP) and dihydropyrimidine dehydrogenase (DPD), together with their possible involvement in promoting 5-FU resistance, however this is yet to be further investigated [206].

##### Apoptosis-Related Pathway

The pro-apoptotic protein apoptosis-activating factor-1 (APAF-1) can also contribute to 5-FU resistance due to being downregulated by miR-23a, which results in inhibition of apoptosis [207]. The expression of miR-23a was significantly increased by 5-FU treatment in both CRC cell lines and CRC human tissues [207]. This research investigation also showed that miR-23a can bind directly to the 3′UTR of Apaf-1, which resulted in the downregulation of APAF-1. Knockdown of miR23a re-sensitized CRC cells and xenografts to 5-FU, with the expression of APAF-1 and caspase 9 also being upregulated. It was eventually concluded that miR-23a regulated 5-FU induced apoptosis via the APAF-1/caspase-9 apoptotic pathways [207]. Chai et al. [208] were the first and only research group to investigate the involvement of miR-20a in CRC chemoresistance. An upregulated expression of miR-20a promoted cellular drug resistance in CRC cell lines by targeting the Bcl-2 family member BCL2 Interacting Protein 2 (BNIP2), which is involved in the mitochondrial-mediated apoptosis pathway [208]. MiR-20a was proven to directly downregulate BNIP2 mRNA and BNIP2 protein levels by binding to BNIP2 3′ UTR to increase 5-FU resistance of CRC cell lines [208]. The study by Zheng et al. [209] demonstrated that miR-9-5p expression is downregulated in CRC cell lines but is then upregulated upon treating CRC cells with 5-FU, together with decreased cell viability. Knockdown of this miRNA decreased the sensitivity of the cells to 5-FU together with apoptotic inhibition, while overexpression of miR-9-5p enhanced sensitivity and induced apoptosis. Furthermore, miR-9-5p also targeted HMGA2, with overexpression of HMGA2 reversing apoptosis triggered by the miRNA itself. In summary, this study showed that the miR-9-5p/HMGA2 axis is associated with 5-FU resistance in CRC cell lines, however further work is required to get a better understanding since this is the only reported study which tackled this phenomenon [209].

The lncRNA Urothelial carcinoma associated 1 (UCA1) can cause 5-FU resistance due to decreasing 5-FU sensitivity of CRC cells via apoptotic attenuation arising by the drug itself [210]. UCA1 was upregulated in CRC tissues and cell lines, as well as in 5-FU resistant cells. Moreover, cells having depleted UCA1 showed a lower survival rate during 5-FU treatment, unlike that observed in the cells having overexpressed UCA1. In addition, this study revealed that the potential molecular mechanism through which UCA1 promoted 5-FU resistance involved UCA1 sponging miR-204-5p and inhibiting its activity, together with upregulating target genes of miR-204-5p (*CREB*, *BCL2*, *RAB22A*) by competitively sponging this miRNA. This work thus presented the first indications for a ceRNA network having UCA1, miR-204-5p and miR-204-5p target genes in CRC resistant cells [210]. In another study, upregulated expression of UCA1 accelerated 5-FU resistance of CRC cells by suppressing apoptosis and enhancing autophagy, via the miR-23b-3p/zinc finger protein 281 (ZNF281) axis [211]. Knockdown of miR-23b-3p reversed the inhibitory effects of UCA1 interference on 5-FU resistance by inhibiting autophagy and promoting apoptotic cell death of CRC cells. Furthermore, miR-23b-3p elevated 5-FU sensitivity via downregulation of ZNF281 in CRC cells, while UCA1 interference also enhanced 5-FU sensitivity. This study concluded that the UCA1/miR-23b-3p/ZNF281 axis mediates 5-FU resistance in CRC, under both in vivo and in vitro conditions [211].

In another study, downregulation of the lncRNA HAND2 Antisense RNA 1 (HAND2-AS1) and upregulation of miR-20a were recognized as being involved in promoting 5-FU resistance in CRC cells [212]. Moreover, overexpression of HAND2-AS1 suppressed resistance of this drug, inhibited cell progression in the resistant cells and promoted cell apoptosis. Thus, this data showed that upregulation of this lncRNA could inhibit 5-FU resistance, by promoting apoptosis [212]. Additionally, HAND2-AS1 acted as a miR-20a sponge, with the expression of both the lncRNA and miRNA being negatively correlated. Decreased miR-20a expression enhanced 5-FU sensitivity, however this was abolished after HAND2-AS1 knockdown [212]. The same study also showed the miR-20a targets PDCD4 to modulate resistance in CRC cell lines. PDCD4 upregulation supported 5-FU sensitivity while this effect was downregulated upon transfecting the cells with agomiR-20a [212]. They concluded that HAND2-AS1 and PDCD4 expression evidently decreased while that of miR-20a markedly increased in 5-FU-resistant CRC. In addition, this was the first study which showed that HAND2-AS1 controls 5-FU sensitivity, inhibits cell progression, and promotes apoptosis by targeting the miR-20a/PDCD4 axis in CRC cells [212]. Qu et al. [213] have recently investigated the role and underlying mechanisms of lncRNA DLGAP1 antisense 1 (DLGAP1-AS1) and hsa-miR-149-5p in the development of 5-FU resistance in CRC cells. Their study shows that the lncRNA was highly expressed in CRC tissues and cell lines, as well as in the treated cells. Furthermore, silencing or overexpression of the lncRNA significantly influenced cell proliferation and the expression of apoptosis related proteins, particularly BCL-2 and proliferating cell nuclear antigen (PCNA). Bioinformatic analysis revealed potential binding sites for DLGAP1-AS1 on miR-149-5p [213]. In fact, wet lab analysis then showed that miR-149-5p overexpression remarkably reduced the expression of DLGAP1-AS1 and that miR-149-5p expression levels markedly increased upon knockdown of the lncRNA, which showed a negative correlation between the two. Further analysis also showed that TGFB2 was also a potential target of this miRNA. Cells having overexpressed wild type TGFB2 exhibited lower miR-149-5p expression, while the opposite was seen in cells having overexpressed miR-149-5p expression since TGFB2 was remarkably repressed in these cells [213]. It was then proven that the lncRNA promotes 5-FU chemoresistance and reduces cell apoptosis by targeting the miR-149-5p-TGFB2 signaling pathway since, when the lncRNA was upregulated, miR-149-5p was downregulated, while TGFB2 was also upregulated. Thus, this study concluded that 5-FU sensitivity in CRC can be modulated via the DLGAP1-AS1/miR-149-5p/TGFB2 axis, however the authors stated that further work has to be carried out to further understand the relationship between DLGAP1-AS1 and TGFB2 as this was not investigated in dept [213].

Xiong et al. showed that circRNAs also contributed to 5-FU resistance via interaction with the BCL2 protein family [201]. Bcl2 was predicted to be regulated by both of the top two upregulated circRNAs in this research investigation; hsa_circ_0007031 and hsa_circ_0000504. However, as previously discussed, further work has to be carried out to confirm most of the assumptions and speculations presented in this study [201]. The circRNA circDDX17 is said to be downregulated in CRC tissues and cells, however its upregulation enhanced 5-FU sensitivity and apoptotic rate in CRC cells [214]. Resistance arose due to this circRNA sponging miR-31-5p, which in turn modulated Kidney Ankyrin repeat-containing protein 1 (KANK1). Thus, overexpression of DDX17 remarkably reduced miR-31-5p expression and KANK1 expression was increased. Ren et al. thus showed that the circDDX17/miR-31-5p/KANK1 axis can modulate the sensitivity of 5-FU in vivo [214]. Despite this being the only reported analysis, the results obtained might provide an opportunity on developing an effective treatment strategy for CRC patients [214].

##### Autophagy

The study by Yu et al. showed that activation of the C-X-C Motif Chemokine 12/C-X-C Motif Chemokine Receptor 4 (CXCL12/CXCR4) axis in CRC resulted in the overexpression of miR-125b [215]. Furthermore, this miRNA conferred resistance to 5-FU in CRC xenografts having overexpressed miR-125b. Resistance was demonstrated to have arisen due to an increased rate of autophagy, as shown by the increased expression of beclin-1 and cleaved LC3-II, as well as increased formation of autophagosomes [215]. MiR-22 is an autophagy related modulator which was found to be downregulated in CRC and in 5-FU resistant CRC cells, however it can also sensitize CRC cells to 5-FU treatment by regulating autophagy and apoptosis [216]. Overexpression of miR-22 significantly enhanced cell death induced by 5-FU in CRC cell lines. In turn, these cells also presented an increase in caspase 7/9 and Poly (ADP-ribose) polymerase (PARP), together with miR-22 significantly inhibiting the expression of TYMS. The opposite was observed when miR-22 was inhibited, with the expression of TYMS increasing and that of caspase 7/9 and PARP decreasing. Despite these results obtained, it was still not clear whether autophagy is involved in 5-FU resistance, so they knocked out autophagy-related gene 5 (ATG5) to interfere with the autophagy induced by miR-22, which resulted in restoration of 5-FU sensitivity [216]. In this study, it was also demonstrated that the mRNA of B-cell translocation gene 1 (BTG1) contained a putative binding site for miR-22 in the 3′-UTR. Cells having overexpressed miR-22 repressed the BTG1 expression not only at the mRNA level but also at the protein level, while upon addition of miR-22 inhibitor, an increased expression of BTG1 was observed. The researchers of the study went on to show that BTG1 increased in cells undergoing autophagy. Thus, upregulation of miR-22 and downregulation of BTG1 increased the sensitivity of CRC cells to 5-FU by inducing apoptosis and inhibiting autophagy. This study revealed a new pathway through which miR-22 regulates autophagy and also highlighted the role of miR-22/BTG1 axis in controlling 5-FU sensitivity [216].

Liu et al. [217] investigated the role of lncRNA nuclear paraspeckle assembly transcript 1 (NEAT1) on cell viability, sensitivity to 5-FU, and autophagy of CRC cell lines. Their study demonstrated an increased NEAT1 expression in CRC cell lines, as well as in the resistant cell lines, while knockdown of this lncRNA resulted in increased 5-FU sensitivity. This study also showed that NEAT1 knockdown can suppress autophagy by targeting miR-34a, as the binding between the lncRNA and the miRNA was discovered via luciferase assay [217]. Cells having knocked out NEAT1 exhibited increased expression of miR-34a while, the opposite was observed in cells overexpressing NEAT1. Furthermore, overexpression of miR-34a downregulated cell proliferation, increased 5-FU sensitivity in the resistant cells and inhibited autophagy due to miR-34a targeting and decreasing the protein expression of High mobility group box 1 (HMGB1), Autophagy Related 9A/B (ATG9A/B). In fact, further analysis showed that miR-34a could potentially have binding sites for the 3 aforementioned proteins. It was then concluded that this was the first study to show that NEAT1 affected the sensitivity of 5-FU and autophagy through the miR-34a/HMGB1/ATG9A/ATG4B axis in CRC cell lines [217].

Another study elucidated that the lncRNA Small Nucleolar RNA Host Gene 6 (SNHG6) was involved in 5-FU resistance by inhibiting apoptosis and promoting autophagy in CRC cells [218]. 5-FU resistant cells exhibited a higher SNHG6 expression than normal untreated cells, while cells having knocked down SNHG6 were more sensitive to 5-FU treatment. Furthermore, the same result was also observed in xenograft models. Additional analyses showed that SNHG6 regulates Unc-51 Like Autophagy Activating Kinase 1 (ULK1) by sponging miR-26a-5p in CRC tissues. In fact, miR-26a-5p was upregulated when SNHG6 was knocked down and downregulated when SNHG6 was overexpressed, while ULK1 was downregulated when miR-26a-5p was overexpressed, while it was upregulated when SNHG6 was overexpressed [218]. Moreover, further analyses revealed that SNHG6 is able to inhibit miR-26a-5p to regulate ULK1-induced autophagy but that miR-26a-5p does not regulate SNHG6. It was concluded that SNHG6 enhances chemoresistance through the ULK1/miR-26a-5p axis [218]. This research was the first in which the involvement of SNHG6 in promoting 5-FU resistance in CRC was investigated.

##### Hippo Signalling Pathway

The study by Xu et al. [219] demonstrated that miR-375-3p is weakly expressed in CRC, while its overexpression restrained the resistance of CRC cells to 5-FU and in turn promotes apoptotic cell death. This phenomenon arose due to Yes-Associated Protein 1 (YAP1) and the transcription factor SP1 being targeted by miR-375, as bioinformatic analysis revealed binding sites on miR-375 for the respective proteins. Both YAP1 and SP1 were upregulated in CRC tissues when compared to normal tissues, with their expression also being higher in 5-FU resistant patients than in 5-FU sensitive patients [219]. Moreover, a significantly negative correlation between YAP1 and SP1 expression and miR-375 was found in CRC tissues. Further investigation demonstrated that overexpression of miR-375 significantly decreased the mRNA expression of YAP1 and SP1, while inhibition of miR-375 remarkably elevated the mRNA expression of YAP1 and SP1 in both parental and 5FU-resistant cell lines. Thus, it was eventually concluded that the miR-375-3p/YAP1/SP1 axis is involved in controlling sensitivity of 5-FU, under both in vivo and in vitro conditions [219].

##### Wnt/β-catenin Signalling Pathway

In the study by Liu et al. [220], miR-149 was significantly downregulated in 5-FU resistant CRC cells when compared to the parental cells. In addition, overexpression of this miRNA resulted in enhanced 5-FU sensitivity, while downregulation increased resistance. Mechanistic investigations revealed that FOXM1 was also a target of miR-149 in the resistant cells. Liu et al. [220] also demonstrated that FOXM1 was upregulated in resistant cells, while its downregulation improved 5-FU sensitivity in drug-resistant CRC cells. Taken together, upregulation of miR-149 could reverse the 5-FU resistance of CRC cells by inhibiting the Wnt/β-catenin signaling pathway due to controlling FOXM1, thus targeting the miR-149/FOXM1 axis may be a promising therapeutic strategy for 5-FU-resistant CRC patients [220]. Another downregulated miRNA which targets FOXM1 in 5-FU resistant CRC cells and in turn slows down the Wnt/β-catenin signalling pathway is miR-320 [221]. Wan et al. [221] demonstrated that enhanced miR-320 expression can inhibit CRC cell proliferation, invasion and increase sensitivity of CRC to 5-FU by targeting FOXM1 and in turn inactivate the Wnt/β-catenin signalling pathway.

In another study, Chen et al. found that 5-FU resistance in CRC tissues and cells resulted in increased expression of circ-PRKDC [222]. In addition, when silencing this circRNA, 5-FU resistance was repressed in the resistant cells, due to circ-PRKDC targeting miR-375. Upon inhibiting miR-375 expression, the suppressive roles of circ-PRKDC interference in drug resistance were weakened, indicating that circ-PRKDC facilitated 5-FU resistance in CRC by modulating miR-375, which also interacted with FOXM1. In fact, the data collected showed that miR-375 could repress 5-FU resistance by targeting FOXM1 in 5-FU-resistant CRC cells [222]. Just like Liu et al. [220], Chen et al. [222] proved that FOXM1 was upregulated in resistant cells, while its downregulation improved 5-FU sensitivity in drug-resistant CRC cells. The Wnt/β-catenin pathway was also modulated by circ-PRKDC, as deficiency of this circRNA decreased β-catenin and c-Myc levels in the 5-FU-resistant CRC cells, which suggested the inactivation of Wnt/β-catenin pathway. This verified that 5-FU resistance in CRC cells is regulated via the circ-PRKDC/miR-375/FOXM1 axis and Wnt/β-catenin pathway [222].

##### Other Chemoresistance Related miRNAs, lncRNAs or circRNAs

Interestingly, a study has shown that miR-375-3p expression is decreased in CRC cells, while when upregulated, it increased 5-FU sensitivity due to targeting TYMS [223]. Overexpression of miR-375-3p decreased the expression of TYMS, promoted apoptosis and cell cycle arrest and inhibited cell proliferation, migration, and invasion. To confirm that TYMS is involved in miR-375-3p mediated 5-FU resistance, the enzyme was silenced and as expected the downregulation of TYMS triggered miR-375-3p-promoted apoptosis and cell cycle arrest, together with inhibited cell proliferation, invasion, and migration. However, further work is required to understand the correlation between miR-375-3p and TYMS, since this is the only reported study which investigated the miR-375-3p/TYMS in 5-FU resistance under in vivo and in vitro conditions [223].

Another research group monitored the expression of NEAT1 in 5-FU resistance CRC cells [224]. Wang et al. were the first to indicate that NEAT1 could regulate 5-FU sensitivity in CRC via the miR-150-5p/Cleavage and Polyadenylation Specific Factor 4 (CPSF4) axis [224]. The data proved that NEAT1 and miR-150-5p expression was negatively correlated (upregulated and downregulated, respectively) in CRC tissues and cells [224]. Knockdown of NEAT1 resulted in increased sensitivity to 5-FU, apoptosis was promoted, and invasion was inhibited by the CRC cells due to NEAT1 regulating CPSF4, which acted as a miR-150-5p sponge [224]. Furthermore, knockdown of NEAT1 resulted in increased miR-150-5p expression, while upregulated NEAT1 expression resulted in miR-150-5p inhibition [224]. To confirm the roles of NEAT1 in controlling CPSF4, it was demonstrated that CPSF4 overexpression overturned the effects of NEAT1 knockdown on the sensitivity of CRC cells to 5-FU treatment [224]. This research investigation showed that the NEAT1/miR-150-5p/CPSF4 axis is responsible for controlling 5-FU resistance in CRC cells [224]. Another recent study also showed that the expression level of NEAT1 is higher in CRC tissues and cell lines when compared to healthy tissues [225]. Once again, NEAT1 expression increased significantly in the resistant cells, while 5-FU sensitivity increased upon knockdown of the lncRNA. Zhu et al. [225] however showed that NEAT1 increased the acetylation of H3K27 in the promoter region of ALDH1 and c-Myc in CRC tissues for patients receiving 5-FU treatment. Thus, their study also demonstrated the involvement of NEAT1 in 5-FU resistance, but through an alternative pathway [225].

Jiang et al. discovered that overexpression of the lncRNA GIHCG in CRC cell lines contributed to cancer progression and 5-FU resistance, but the mechanisms of GIHCG promoting such progression and resistance remains unknown, as this was not further investigated [226]. Their study showed that GIHCG is typically upregulated in CRC cell lines, human tissues, and tumour tissues. Additionally, resistant cells had a higher expression of this lncRNA, while when downregulated the cell survival decreased. The data presented in this study was the first to reveal the function of GIHCG in chemoresistance, however further work has to be carried out to further understand the role of GIHCG and how it contributes towards promoting resistance [226]. Furthermore, the lncRNA Taurine Upregulated Gene 1 (TUG1) was also shown to be associated with 5-FU resistance in CRC [227]. Its overexpression promoted proliferation of 5-FU resistant cells while knockdown re-sensitised the resistant cells to 5-FU. This study revealed interactions between miR-197-3p and TUG1, with TUG1 also regulating the TYMS enzyme by acting as a ceRNA to sponge miR-197-3p. This interaction promoted decreased miR-197-3p expression and increased TYMS expression, which in turn gave rise to resistance. Thus, it was concluded that 5-FU resistance in CRC could be modulated via the TUG1/miR-197-3p/TYMS axis. This study, for the first time, revealed that TUG1 was upregulated in CRC recurrence tissues and 5-FU resistant cell lines, with the findings of the study highlighting the potential value of TUG1 as a predictive biomarker [227]. The study by Qiao et al. [228] showed that 5-FU sensitivity can increase in CRC cell lines which have undergone knockdown of the lncRNA prostate cancer-associated ncRNA transcript 1 (PCAT-1). The expression of this lncRNA was upregulated in CRC tissues and cells resistant to 5-FU. However, 5-FU treatment led to significant increases in the early and late apoptotic rates in PCAT-1-silenced cells as compared to the parental CRC cells. Since this was the only study which monitored the role of PCAT-1 in CRC cell lines as they gained chemoresistance, further work has to be done so as to reveal the molecular mechanisms underlying the functions of this lncRNA in 5-FU resistance [228].

Xiong et al. were the first to discover 71 circRNAs differentially expressed in 5-FU- and radiation-resistant CRC cells via microarray analysis [201]. Among these circRNAs, 47 were upregulated and 24 were downregulated, with hsa_circ_0007031, hsa_circ_0007006 and hsa_circ_000504 being the three most upregulated, while hsa_circ_0008509, hsa_circ_0084021 and hsa_circ_0087862 were the three most downregulated. Furthermore, bioinformatic analysis (using KEGG pathways) revealed that modulated circRNAs in 5-FU chemoradiation-resistant CRC cells are involved in mediating several miRNAs and cancer related signalling pathways, such as the actin-cytoskeleton pathway, focal adhesion signalling, and WNT signalling pathway, all of which are associated with CRC development [201]. They also proposed that miR-885-3p and hsa_circ_0007031 target each other and since the latter circRNA was found to be highly upregulated in this study, they suggested that hsa_circ_0007031 might play a crucial role in the development of resistance [201]. Furthermore, hsa_circ_0000504 which was also upregulated in this study was shown to control the interaction between the Signal transducer and activator of transcription 3 (STAT3) protein involved in the JAK/STAT signalling pathway and hsa-miR-485-5p. They suggested that upregulation of hsa_circ_0000504 could reduce the suppression of hsa-miR-485-5p on STAT3 and accelerate the development of 5-FU resistance in CRC. Thus, they also speculated that by downregulating hsa_circ_0000504, it could be possible to overcome 5-FU resistance in CRC [201]. Among the downregulated circRNAs, it was found that hsa_circ_0048234 has four miR-671-5p-binding sites and was previously modulated in cetuximab and panitumumab resistance CRC cell lines [229]. Ragusa et al. [229] had previously discovered the interaction between miR-671-5p and the EGFR signaling pathway and its roles in chemoresistant CRC cell lines, so Xiong et al. [201] proposed that the downregulation of hsa_circ_0048234 could increase EGFR signaling and promote CRC resistance by targeting miR-671-5p. Despite being the first research group to provide a database for understanding the role of differentially expressed circRNAs in 5-FU resistant CRC cell lines, further work has to be carried out to confirm most of the assumptions and speculations presented in this study [201]. In a more recent study, which tackled a similar investigation, Abu et al. sought to identify differentially expressed circRNAs between 5-FU and OXA (FOLFOX) resistant and chemosensitive CRC cells [230]. From their analysis, 773 upregulated and 732 downregulated circRNAs were identified between the resistant CRC HCT116 cell line and the parental cell line, with the exonic has_circRNA_103306 being the most upregulated and the exonic has_circRNA_406937 being the most downregulated [230].

A significant upregulation of circ_0032833 was present in CRC cells which were resistant to FOLFOX [231]. However, upon circ_0032833 knockdown, the FOLFOX-resistant CRC cells were more sensitive to 5-FU and OXA. Moreover, circ_0032833 acted as an miR-125-5p sponge and regulated Musashi1 (MSI1), which resulted in the FOLFOX-resistant CRC cells being more susceptible to 5-FU and OXA. Thus, Li and Zheng were the first to report that the circ_0032833/miR-125-5p/MSI1 axis can regulate 5-FU and OXA sensitivities in CRC cells/tissues resistant to FOLFOX, both under in vitro and in vivo condition [231].

#### 3.2.2. miRNAs, lncRNAs, circRNAs in OXA Resistance

OXA resistance is another problem which researchers are trying to further understand. Resistance to this drug is achieved due to different changes occurring in the cell, including reduced drug uptake and/or enhanced efflux of the drug, reduced response to the platinum DNA adducts, EMT, decreased DNA adduct formation due to mutations in certain proteins involved in NER as explained previously, increased DNA repair, increased adduct tolerance and modulation of the cell death pathways [17,18,232,233]. Something to note is that unlike most drugs which commonly have a direct effect on specific molecules/genes typically involved in particular signalling pathways to give rise to resistance in CRC, OXA acts indirectly via numerous alternate pathways. In fact, there have been only limited studies showing that OXA resistance can arise due to a direct dysregulation of molecules/genes involved in for instance the PI3K/AKT pathway [234]. Most studies have however shown that OXA acts on the signalling pathways indirectly (or via an alternative pathway) by targeting other molecules which can communicate with the targets involved in said signalling pathways [235,236,237]. In addition to this, miRNAs, lncRNAs and circRNAs have also shown to target specific pathways not commonly involved in CRC OXA resistance, as will be discussed below (Table 2). Furthermore, all the three ncRNAs have been shown to be involved in the majority of the aforementioned mechanisms of resistance (Table 2).

##### ABC Transporters Family

The main aim of the study by Gao et al. was to investigate the functional role of cancer susceptibility candidate 15 (CACS15) in CRC OXA resistance as well as its underlying molecular mechanism. They demonstrated that OXA resistance can develop in CRC cells due to this lncRNA being overexpressed in these cells, while its silencing resulted in increased OXA sensitivity in the resistant cells [238]. The phenomenon arose due to CACS15 acting as a ceRNA, decreasing the expression of ABCC1 via miR-145 sponging. Thus, this study revealed that OXA resistance in CRC cells can be modulated via the CACS15/miR-145/ABCC1 axis [238].

##### PI3K/AKT Signalling Pathway

In a study by Yue et al., LINC00152 was shown to promote tumor progression and confer CRC resistance to oxaliplatin in both in vitro and in vivo conditions [239]. LINC00152, which was upregulated, promoted OXA resistance in CRC by acting as a ceRNA and sponging miR-193a-3p [239]. LINC00152 modulated the expression of Erb-B2 Receptor Tyrosine Kinase 4 (ERBB4) which can activate the PI3K/AKT pathway by sponging this miRNA. This led to the activation of AKT, which further contributed to resistance in the CRC cells. When ERBB4 was downregulated, AKT phosphorylation was downregulated and resistance also decreased, which indicated the involvement of the Linc00152/miR-193a-3p/ERBB4/AKT signalling axis in regulating OXA resistance in CRC [239]. In the study by Li et al. [240] OXA resistant CRC cells presented a significantly high expression of the oncogenic circRNA CCDC66. Knockdown of this circRNA downregulated genes which are controlled by circCCDC66-associated miRNAs and others which are responsible for controlling cell cycle and apoptosis. This resulted in cell survival suppression and oxaliplatin-induced apoptosis, thus preventing resistance from arising. The increased expression of circRNA CCDC66 was induced by oxaliplatin via PI3K-mediated DHX9 phosphorylation [240]. Thus, Lin et al. proposed that circRNA CCDC66 induction dependent on oxaliplatin treatment and DHX9 is required for oxaliplatin resistance to arise in CRC cells [240].

##### Apoptosis-Related Pathway

Apart from promoting 5-FU resistance, miR-20a has been shown to also contribute to OXA resistance. In fact, in the same study carried out by Chai et al. [208] where they investigated 5-FU resistance due to miR-20a, their results also showed that overexpression of miR-20a due to OXA also modulates BNIP2 expression, resulting in resistance due to blockage of events leading to apoptosis [208]. In another study, in vivo and in vitro investigation showed that increased expression of miR-153 was not only responsible for increased CRC growth but also gave rise to OXA and cisplatin resistance by blocking the Forkhead transcription factor ‘Forkhead box O3a’ (FOXO3a), responsible for a variety of processes, particularly initiation of the apoptotic pathway [241]. The apoptosis related gene programmed death protein 10 (PDCD10) was shown to be modulated by miR-425-5p in the development of OXA and 5-FU resistant CRC cells [242]. Expression of miR-425-5p was significantly upregulated in resistant cell lines compared to the parental cells. However, inhibition of miR-425-5p reversed chemoresistance in the cells. In addition, for this phenomenon to arise, the miRNA has to regulate the PDCD10 in both in vivo and in vitro conditions [242]. Qin et al. [243] investigated how miR-135b is involved in promoting resistance in CRC. This miRNA was overexpressed in both CRC cell lines and serum from patients suffering from CRC, and in turn also gave rise to OXA resistance. However, upon miR-135b knockdown, CRC cells were sensitised to OXA induced cytotoxicity. MiR-135b knockdown also increase the expression of Forkhead box 1 (FOXO1) in the cells and increased the sensitivity of OXA resistant cells [243]. The miR-135b/FOXO1 axis controlled the pro-apoptotic proteins Bim and Noxa, with their expression increasing in cells having miR-135b inhibitors, which in turn promoted mitochondrial apoptosis. In conclusion, this research group was the first and only group who showed that knockdown of miR-135b enhances efficacy of oxaliplatin-based treatment in CRC by targeting FOXO1 [243].

One very recent study conducted by Chen et al. [244] sought to investigate and uncover the role and potential mechanism of the lncRNA bladder cancer-associated transcript 1 (BLACAT1) in CRC progression and OXA resistance. The expression of BLACAT1 was upregulated in CRC tissues and cells and in OXA resistant cells. Upon silencing of this lncRNA, the resistant cells were resensitized to OXA and apoptosis was further facilitated, while cell proliferation was inhibited. Then when overexpressed, BLACAT1 promoted resistance. The study went on to show that BLACAT1 can regulate miR-519d-3p due to the miRNA having complementary sequences with this lncRNA. MiR-519d-3p expression was enhanced by BLACAT1 deletion, but it was downregulated when BLACAT1 was upregulated, thus a negative correlation was observed between the two. Furthermore, miR-519d-3p overexpression or BLACAT1 knockdown promoted the expression of cleaved-caspase-3 and inhibited the expression of Matrix metallopeptidase 9 (MMP-9). In this study, the researchers also demonstrated an interaction between miR-519d-3p and CAMP Responsive Element Binding Protein 1 (CREB1) which is an oncogene responsible for controlling cell proliferation and differentiation. Additionally, overexpression of the miRNA resulted in significant decrease of CREB1 expression, while anti-miR-519d-3p significantly increased CREB1 expression. Furthermore, BLACAT1 deletion suppressed CREB1 expression, which was reversed with miR-519d-3p inhibition [244]. This study was the first to reveal the functional roles of BLACAT1 in suppressing apoptosis and controlling OXA sensitivity in CRC cells by targeting miR-519d-3p which in turn controls CREB1 expression in CRC progression under in vitro conditions. The limitation of this study was that the interaction between miR-519d-3p and BLACAT1 or CREB1 was initially identified via dual-luciferase reporter assay and should be verified by RNA pull-down or RNA immunoprecipitation. In addition, the data collected, and the conclusions set using commercial cell lines could not fully represent the actual situation in vivo [244].

Lastly, studies have shown that the lncRNA maternally expressed gene 3 (MEG3) is downregulated in OXA resistant CRC cells, and upregulation of MEG3 expression reversed OXA resistance in CRC cell lines [245,246]. In addition, Wang et al. also discovered that MEG3 overexpression enhanced the sensitivity of CRC cells to OXA by upregulating PDCD4, that sponges miR-141. This is because miR-141 was found to be a potential target of MEG3, and in fact overexpression of the miRNA resulted in decreased expression of the lncRNA. Furthermore, the miRNA was significantly upregulated in the OXA resistant CRC tumour tissues when compared to normal colon tissues and sensitive OXA tissues [246]. Moreover, overexpression of MEG3 resulted in downregulation of the miRNA and increased OXA sensitivity for the resistant CRC cells. It was also proven that the miRNA could interact with PDCD4, with the expression of this protein being repressed in cells having overexpressed miR-141. In conclusion, the data collected showed that the MEG3/miR-141/PDCD4 regulatory axis could overcome OXA resistance in CRC, under both in vivo and in vitro conditions [246].

##### Autophagy Related Pathway

In the study by Ta et al. [247], a negative correlation was observed between miR-409-3p expression and OXA resistance in CRC cells and tissues. The miR-409-3p expression levels were lower in human colon cancer patient samples than in normal colon tissues as well as in the cell lines. OXA resistant cells exhibited significantly downregulated miR-409-3p levels, but higher autophagic activity than the OXA sensitive cells. Bioinformatic analysis revealed an interaction between miR-409-3p and Beclin 1. The data collected indicated that the overexpression of miR-409-3p inhibited Beclin-1 expression and autophagic activity by binding to the 3’-untranslated region of Beclin-1 mRNA, which enhanced the chemosensitivity of the OXA sensitive and OXA resistant CRC cells [247]. The same phenomenon was also observed under in vivo conditions, as the tumour was more sensitive to OXA. Despite being the only study, which investigated miR-409-3p/Beclin 1 in OXA resistance, the findings suggest that this miRNA can enhance the chemosensitivity of colon cancer via Beclin 1 mediated autophagy inhibition [247].

Yanli et al. [248] where the first to report an increased expression of circHIPK3 in OXA resistant CRC cell lines, CRC patient tissues and xenografts. Moreover, knockdown of this circRNA resensitised the OXA resistant cells to the drug. Furthermore, circHIPK3 functioned as an efficient miR-637 sponge, with this miRNA proven to interact with STAT3. In this study, activation of STAT3 was inhibited by miR-637, resulting in downregulation of Bcl-2, thus increasing beclin 1 due to its release from the Bcl-2-beclin-1 complex, to initiate autophagy. In addition, a positive relationship was detected between circHIPK3 and STAT3, which hinted that circHIPK3 could serve as a ceRNA by sponging miR-637 to trigger the STAT3 signalling pathway, thus enhancing Bcl-2 expression and blocking beclin1 dissociation. Eventually this led to reduced autophagic cell death which contributed to OXA resistance [248]. This provides a promising prognostic predictor in CRC patients being treated with OXA.

##### Wnt/β-catenin Signalling Pathway

In the same study carried out by Wan et al. [221], were they showed that miR-320 expression can inhibit CRC cell proliferation, invasion, and increase sensitivity of CRC to 5-FU by targeting FOXM1 and in turn inactive the Wnt/β-catenin signalling pathway, they also showed that similar results are obtained in OXA resistant CRC cells. In another study, Ren et al. [249], investigated one of the most studied lncRNAs H19, which is typically upregulated in CRC cells, so as to investigate its role in OXA resistance when using CRC cell lines and xenografts. H19 was highly expressed in the tumor tissues when compared to that of normal colon tissues. Overexpression of lncRNA H19 contributed to OXA resistance in different CRC cell lines and xenografts [249]. In addition, their data shows that resistance arose partly due to the lncRNA H19 activating the β-catenin pathway by acting as a competing endogenous RNA sponge for miR-141 [249].

##### TNF-α Pathway

OXA resistance in CRC cell lines developed due to upregulation of hsa_circ_0079662 expression, according to Lai et al. [250]. The drug resistant roles of hsa_circ_0079662 arose due to it sponging hsa-miR-324-5p, which led to downregulation of this miRNA, together with the activation and upregulation of Homeobox A9 (HOXA9) and upregulation of TNF-α, IL-1 and IL-6 [250]. This study was the first to shown that the hsa_circ_0079662/hsa-mir-324-5p/HOXA6 axis induces OXA resistance in in vitro conditions and xenograft models via the TNF-α pathway in CRC [250]. If further validated, the hsa-miR-324-5p may be a potential molecular marker with promising application perspectives for CRC.

##### Glycolysis

The circular RNA hsa_circ_0005963 was proven to be upregulated in OXA resistant CRC cells [251]. It was further demonstrated that this circRNA acts as a miR-122 sponge targeting and upregulating pyruvate kinase (PKM2) and in turn gives rise to OXA resistance in CRC cells. In addition, extraction of exosomes from these resistant cells transferred to OXA sensitive cells, enhanced glycolysis and promoted resistance by upregulating the expression of PKM2 [251]. Thus, Wang et al. showed that OXA resistance in CRC cells can arise via the hsa_circ_0005963/miR-122/PKM2 axis [251].

##### Other Chemoresistance Related miRNAs, lncRNAs or circRNAs

In the previously discussed research investigation by Jiang et al. [226], in which they showed that 5-FU resistance arose due to overexpression of the lncRNA GIHCG, their data also showed that the same phenomenon can arise for CRC cells treated with OXA. However, as already pointed out, the mechanism involved in giving rise to such resistance was not further investigated [226]. Zhou et al. [252] were the first to report OXA resistance due to miR-203. In silico analysis indicated that ataxia telangiectasia mutated (ATM) kinase, a primary mediator of the DNA damage response, was possibly a target of miR-203. MiR-203 was upregulated in OXA resistant CRC cells while upon its knockdown, the resistant cells were resensitised to OXA. Furthermore, ATM was significantly downregulated in CRC cells with acquired resistance to OXA. Zhou et al. [252] went on to show that miR-203 mediates suppression of ATM in the resistant cells, with the data collected being the first such report.

In another study, Liang et al. showed that OXA resistant CRC cells had downregulated miR-483-3p, which was concurrent with upregulated Family With Sequence Similarity 171 Member B (FAM171B) [253]. Inhibition of this miRNA enhanced OXA resistance in the CRC cell lines, while its overexpression increased OXA sensitivity. This research groups showed that miR-483-3p regulated OXA resistance by targeting FAM171B. In fact, FAM171B levels decreased in OXA resistant CRC cells transfected with miR-483-3p, while FAM171B expression increased after miR-483-3p inhibitor treatment. In conclusion, this was the first study supporting an association between miR-483-3p, FAM171B and OXA resistance in CRC, thus further efforts are required to test the effect of miR-483-3p adjuvant treatment in clinical use combined with OXA for cancer patients [253]. In a similar study, Peng et al. demonstrated that exogenous expression of miR-492 in OXA resistant CRC cells could confer OXA treatment sensitivity, together with a decreased expression of CD147 [254]. This study hinted that the miR-492/CD147 is linked with OXA resistance in CRC [254]. The miR-200b-3p was downregulated in OXA resistant CRC tissues and cells, while when overexpressed, OXA sensitivity was increased in the resistant cell lines [255]. This research group determined the potential mechanism involved in promoting OXA resistance, with their data showing that miR-200b-3p mediated reversal of OXA resistance by controlling its downstream target β-III tubulin (TUBB3) [255]. When the miRNA was overexpressed, growth inhibition and apoptosis were induced in the resistant cells, with the expression of TUBB3 being downregulated. On the other hand, overexpression of TUBB3 inhibited miR-200b-3p, growth inhibition and apoptosis in OXA resistant CRC cells. This study was the first reported analysis which investigated the involvement of the miR-200b-3p/TUBB3 axis in modulating OXA sensitivity in CRC [255].

The lncRNA LINC00460 was upregulated in OXA resistant CRC cell lines when compared to parental OXA sensitive cell lines [256]. This study showed that this lncRNA was responsible for OXA resistance in cells having mutated p53. Using bioinformatic analysis, it was revealed that LINC00460 can interact and target miR-149-5p and miR-150-5p, both of which exhibited lower expression in the resistant cells, while they were upregulated upon silencing of the lncRNA. Additionally, the p53 protein was predicted to be a potential target for the two miRNAs, with its expression decreasing in cells having silenced LINC00460 and overexpression of the two targeted miRNAs. Their data showed that LINC00460 must have promoted OXA resistance by competitively binding to miR-149-5p/miR-150-5p and upregulating the expression of the corresponding miRNA target p53, when under in vitro conditions [256]. Furthermore, the mutated p53 protein level was positively correlated with LINC00460, thus they speculated that the expression of the mutated p53 would be altered upon LINC00460 knockdown. They also hypothesised that p53 might regulate the expression of LINC00460 due to the lncRNA having a promoter region for the p53 transcription factor. In fact, this hypothesis was then proven due to LINC00460 expression being downregulated in cells having p53 knockdown. Collectively, this study concluded that OXA can trigger LINC00460 in the cytoplasm which functions as a ceRNA by targeting miR-149-5p/miR-150-5p and upregulating the expression of the miRNA target, mutant p53 and in turn give rise to OXA resistance in CRC [256].

In another recent study, the lncRNA CBR3-AS1 was overexpressed in OXA resistant CRC cells, and upon knockdown of this lncRNA, it significantly enhanced OXA sensitivity in the resistant cells [257]. Xi et al. provided evidence that this lncRNA acts as an miR-145-5p sponge and through this interaction, it is responsible for promoting OXA resistance and stem-like properties in CRC cells [257]. Compared to the parental cells, miR-145-5p expression was also decreased in OXA resistant CRC cells. It was concluded that the data collected was convincing enough to show that CBR3-AS1 promotes stem-like properties and OXA resistance in CRC cells by sponging miR-145-5p [257]. Another recent study demonstrated that lncRNA metastasis associated lung adenocarcinoma transcript 1 (MALAT1) was upregulated in OXA resistant CRC, while its deficiency resulted in enhanced OXA sensitivity in the resistant cells [258]. Further analysis showed that MALAT1 can directly interact with miR-324-3p, with the expression of the miRNA being downregulated when the lncRNA was upregulated and also in the OXA resistant cells. Apart from the interaction between the lncRNA and miRNA, the miRNA was shown to also interact with a disintegrin and metalloprotease metallopeptidase domain 17 (ADAM17), the mRNA and protein levels of ADAM17 being upregulated in the OXA resistant tissues and cells, which hinted to its role as a mediator in CRC chemoresistance [258]. In fact, upon further investigation it was concluded that MALAT1 exerted promotion effects on OXA resistance via the miR-324-3p/ADAM17 axis for both in vitro and in vivo conditions [258]. Nevertheless, since this is the only published study, further work and exploration is suggested.

#### 3.2.3. miRNAs, lncRNAs, circRNAs in Cisplatin Resistance

Since cisplatin and OXA come from the same family of drugs, it can be assumed that the mechanisms of resistance arising for these drugs are similar and related. However, this is not always the case as mechanisms of resistance for OXA tend to be slightly different to those of cisplatin as explained in the previous section. The main mechanisms of resistance for cisplatin in CRC include, ROS signalling pathways, loss of MMR, upregulation of certain drug transporters such as MRP2, BCRP and SLC among others, as well as apoptotic and autophagic pathways [17,18,259,260]. Just like OXA, there have been limited studies which have shown that cisplatin resistance can arise due to cisplatin directly targeting and dysregulating different molecules/genes involved in different signalling pathways. However, cisplatin is said to target signalling pathways such as the Wnt/β-catenin signaling pathway, using alternative ways just like OXA [261]. In addition, this drug has also shown to affect certain ncRNAs which can in turn target specific molecules in different signalling pathways and give rise to resistance as will be discussed below (Table 3). To our knowledge, there have been no reports to date which tackled cisplatin resistance in CRC arising due to circRNAs.

##### ABC Transporters Family

Peng et al. [262] investigated the role of PVT1 in cisplatin resistant CRC cells for both in vitro and in vivo conditions Similar to the results published by Fan et al. [198], PVT1 was highly expressed in CRC tissues and cell lines compared to the corresponding non-cancerous samples and normal colon epithelial cells. However, this study demonstrated that PVT1 gave rise to cisplatin resistance due to upregulation of the expression of MRP1 and MDR1 and by blocking the intrinsic apoptotic pathway. Upon PVT1 knockdown in the cisplatin-resistant CRC cells, proliferation was inhibited, and the transport pumps were downregulated. In addition, the lncRNA also had an effect on certain apoptotic proteins as will be discussed in the apoptosis related pathway section. This study being to only one to investigate this phenomenon in CRC showed that by targeting PVT1, it was possible to provide better insights for effective CRC therapy [262].

##### Apoptosis-Related Pathway

As explain previously, the lncRNA PVT1 contributed to cisplatin resistance not only through ABC transport pumps, but also by targeting apoptosis [262]. Overexpression of PVT1 was shown to enhance cisplatin resistance by inhibiting intrinsic apoptosis of CRC cells [262]. The anti-apoptotic protein BCL-2 was also upregulated in the resistant cells, while the pro-apoptotic BAX and cleaved caspase-3 were downregulated, thus PVT1 inhibits the intrinsic apoptotic pathway in cisplatin resistant CRC cells [262]. On the contrary, Ping et al. also showed that cisplatin resistance is reversed if PVT1 is inhibited, with the expression of Bax and cleaved caspase-3 being reversed too [262]. In another study, Zheng et al. showed that the lncRNA KCNQ1 Opposite Strand/Antisense Transcript 1 (KCNQ1OT1) is significantly upregulated in cisplatin resistant CRC cells, while cisplatin sensitivity was enhanced if KCNQ1OT1 was silenced [263]. They demonstrated that cisplatin resistance arose due to KCNQ1OT1 targeting and downregulation of miR-497 expression, which in turn removed the suppressive effect of this miRNA on the anti-apoptotic protein Bcl-2. Thus, it was concluded that the KCNQ1OT1/miR-497/Bcl-2 axis was a contributor to cisplatin resistance in CRC cells for both in vivo and in vitro conditions. However, this was the only study carried out on CRC to investigate such circumstances [263].

##### Autophagy

Han et al. were the first to report that overexpression of the lncRNA Small Nucleolar RNA Host Gene 14 (SNHG14) played a role in the development of cisplatin resistance in CRC tumour cells and tissues via autophagy [264]. Their data showed that SNHG14 and the autophagy protein ATG14 were upregulated in CRC tumour tissues when compared to normal ones, while miR-186 is downregulated. Bioinformatic and luciferase reporter assays revealed an interaction between SNHG14 and miR-186. SNHG14 could directly interact with miR-186 and inhibit its expression. Meanwhile, miR-186 could directly bind ATG14 to inhibit its expression level. In cisplatin resistant cells, overexpression of ATG14 significantly enhanced the cell proliferation rate and inhibited cell apoptosis. In conclusion, this research group was the first to establish a novel axis of SNHG14/miR-186/ATG14 in CRC cells which could be pivotal in regulating CRC development and cisplatin resistance in CRC cells and tissues [264].

##### Wnt/β-catenin Signaling Pathway

The study by Xiao et al. demonstrated that miR-203a-3p was downregulated in cisplatin resistant CRC cells [265]. Overexpression of this miRNA sensitised the CRC cells and tissues to cisplatin by inhibiting the Wnt/β-catenin signalling pathway upon targeting β-catenin and Groucho related gene 5 (GRG5), two molecules which are found downstream of this pathway. In addition, they also showed that the lncRNA HOX Transcript Antisense RNA (HOTAIR) is overexpressed in the resistant cells and tissues. However, HOTAIR was also said to be downregulated by miR-203a-3p. Furthermore, knockdown of HOTAIR and overexpression of miR-203a-3p inhibited the Wnt/β-catenin signaling and proliferation of CRC cells, together with increased sensitivity to cisplatin. This was the first study on such a phenomenon, in which they concluded that lncRNA HOTAIR controls the progression and chemoresistance of CRC by targeting miR-203a-3p and the Wnt/β-catenin signaling pathway [265]. In another study by Wang et al. [266], their data showed that LINC00261 can sensitise cisplatin-resistant CRC cells by enhancing apoptosis through decreased Wnt/β-catenin pathway activation. The data collected showed that this lncRNA is downregulated in both colon cancer cell lines and tissues, and in cisplatin-resistant cells. When overexpressed, LINC00261 sensitised the resistant cells to cisplatin due to increased apoptotic cell death, inhibited cell viability, invasion, and migration. Upon further analysis, this phenomenon was shown to have arisen due to LINC00261 downregulating nuclear β-catenin by restraining the transit of β-catenin from the cytoplasm into the nucleus or by promoting β-catenin degradation and inhibiting Wnt activation [266].

##### EMT

Another study conducted by Ren et al. showed that miR-514b-3p was downregulated in CRC cells and tissues, while miR-514b-5p was upregulated [267]. Furthermore, the data revealed that cells with overexpressed miR-514b-5p had increased viability compared to those treated with control miRNA, and there was no significant difference in the absence of cisplatin, while overexpression of miR-514b-3p accelerated cell death in cells treated with cisplatin. To further verify this for in vivo conditions, xenograft mice were established with miR-514b-3p or miR-514b-5p lentivirus. No change in tumour size was observed in the absence of cisplatin, but under treatment, mice having the miR-514b-3p showed a decreased tumour size, while an increased tumour size was observed in the mice having miR-514b-5p. In addition, they investigated whether EMT was involved in the process, with their data showing that the epithelial markers E-cadherin and CLDN-1 increased at both mRNA and protein levels after ectopic miR-514b-3p expression, while the mesenchymal markers fibronectin-1 and vimentin decreased. However, the opposite was seen with miR-514b-5p, thus they concluded that miR-514b-3p could suppress, while miR-514b-5p could promote tumor metastasis by regulating EMT. However, further work has to be carried out on how these miRNAs are involved in possibly controlling cisplatin resistance via EMT, as this is the only study carried out to date [267].

##### Other Chemoresistance Related miRNAs or lncRNAs

Zhang et al. explored the effects of miR-20a on cisplatin treatment in CRC [268]. Their study demonstrated increased expression of miR-20a in CRC cells when compared to normal colon cells. The data generated showed that miR-20a negatively regulated cisplatin sensitivity in CRC under both in vitro and in vivo conditions. In fact, sensitivity increased once miR-20a was knocked down. Further analysis revealed that miR-20a regulated cisplatin sensitivity by controlling the ROS signalling pathway and by targeting apoptosis signal-regulating kinase 1 (ASK1) and c-Jun N terminal kinase (JNK) [268]. They concluded that miR-20a can decrease cisplatin sensitivity in CRC cells, but knockdown of miR-20a could improve the sensitivity of CRC cells to cisplatin via the ROS/ASK1/JNK pathway [268].

The lncRNA differentiation antagonising non-coding RNA (DANCR) was investigated by Shi et al. to determine its involvement in regulating cisplatin resistance in CRC [269]. DANCR was upregulated in both CRC tissues and in cisplatin resistant CRC cells. In addition, its overexpression desensitised the colon cells to cisplatin, while this was reversed when this lncRNA was silenced. Further analyses revealed that DANCR acted as a ceRNA, being able to bind to miR-125b-5p, with the correlation between the two being negative since miR-125b-5p was downregulated when DANCR was overexpressed [269]. Moreover, overexpression of miR-125b-5p enhanced the sensitivity of cisplatin resistant cells. Apart from these observations, the same study also noted an increase in glycolysis rate in the resistant cells, due to miR-125b-5p targeting the hexokinase 2 (HK2) enzyme in these cells. Shi et al. went on to show a new mechanism through which cisplatin resistance developed in the colon cells, via the DANCR/miR-125b-5p/HK2 axis [269]. Another lncRNA which was shown to be upregulated in cisplatin resistant CRC cells is MIR4435-2 Host Gene (MIR4435-2HG) [270]. When MIR4435-2HG was silenced, it enhanced the sensitivity of cisplatin resistant CRC cells, together with inhibiting cell proliferation and promoting apoptotic cell death. To perform such roles, Luo et al. were the first to report that MIR4435-2HG modulated cisplatin resistance by targeting Nuclear factor erythroid 2-related factor 2 (Nrf2) and heme oxygenase-1 (HO-1), both of which are related to oxidative stress [270]. In addition, when silencing MIR4435-2HG, mRNA levels of HO-1 and Nrf2 were also downregulated, especially after treatment with cisplatin, which hinted at MIR4435-2HG involvement in oxidative stress, concluding that the lncRNA MIR4435-2HG could contribute to cisplatin resistance by targeting the Nrf2/HO-1 pathway [270].

#### 3.2.4. miRNAs, lncRNAs, circRNAs in DOX Resistance

Despite DOX not being considered a first line treatment for patients suffering from CRC, it was still shown to be suitable for adjuvant chemotherapy at advanced stages of CRC [271]. Even though it has been proven to work on CRC [271,272], one of the main reasons why DOX is not commonly used to treat CRC is due to the resistance arising at early stages. Over expression and upregulation of ATP membrane transporters are one of the most understood mechanisms of DOX resistance, in not only CRC but also in other cancers [18,273]. However, DOX resistance can also arise due to EMT, some signalling pathways which include the MAPK/ERK and AKT/PI3K pathways, cell death pathways (apoptosis and autophagy) and due to specific proteins (e.g., p53, Type IIA topoisomerases, and estrogen receptor alpha), as reviewed by Micallef et al. [16]. LncRNAs and miRNAs have been shown to promote or decrease DOX resistance in CRC by targeting specific proteins in apoptosis, EMT, certain ABC transporters, PI3K/AKT, MAPK/ERK and Notch signalling pathways (Table 4). However, to our knowledge there have not been any reports to date which tackled DOX resistance in CRC arising due to circRNAs.

##### ABC Transporters Family

Yang et al. [274] were the first and only research group to investigate miR-522 in DOX resistant CRC cell lines. Their study showed that the miR-522 expression decreased significantly in the DOX resistant cell lines when compared to the parental untreated cell line. However, upon overexpressing miR-522 in the resistance cells, DOX sensitivity was partially restored. Further investigation showed that this miRNA could reverse the resistance in the cell lines by targeting one of the ATP-binding cassette protein pumps, ABCB5, with the expression of the two being inversely correlated. ABCB5 knockdown increased growth inhibition and improved DOX sensitivity in the resistant cells, which suggested that DOX resistance in CRC cell lines can be modulated by miR-522, which decreases the expression of the ABCB5 pump. This study thus demonstrated and concluded that miR-522 decreased cell survival and DOX resistance in human CRC by directly targeting ABCB5 [274]. In a more recent study, miRNA-29a was also proven to be involved in modulating DOX resistance in CRC cells [275]. Shi et al. showed that the expression level of miR-29a was higher in the parental CRC cell lines than in the DOX resistant cells [275]. However, they further demonstrated miR-29a overexpression could reverse DOX resistance in resistant CRC cells, while downregulation of this miRNA could promote the development of DOX resistance. In addition, miR-29a overexpression decreased the expression and activity of MDR1/P-gp which contributed to increased DOX sensitivity in the resistant cells. However, they speculated that miR-29a could play a crucial role in the inhibition of MDR1/P-gp expression by down-regulating PI3K/Akt signaling pathway activity by targeting and upregulating the PTEN enzyme, which is a negative regulator of the PI3K/AKT pathway. This study provided insight into the role of the miR-29a/PTEN/PI3K/Akt/MDR1/P-gp axis in CRC DOX resistance [275]. Despite this study being the only one to have investigated DOX resistance in CRC due to miR-29a, the data showed that interference of miR-29a expression could be potentially useful for the prediction of the clinical response, providing a promising target for the treatment of CRC [275].

##### PI3K/AKT, MAPK/ERK Signalling Pathways and EMT

A recent study has investigated the role of miR-223 on FBXW7 in CRC and obtained similar results for the expressions of miR-223 and FBXW7 in non-treated CRC cells [276]. However, they suggested that the Notch pathway and AKT/mTOR pathway are regulated by the miR-223/FBXW7 axis, while no investigation was carried out on EMT markers [276]. Despite their data, further work has to be carried out to determine how such pathways are controlled by the miR-223/FBXW7 axis in DOX resistant cells, since this was not part of either research investigation.

The signal transduction protein, extracellular signal-regulated kinase 1 (ERK1) which forms part of the ERK/MAPK pathway was shown to be regulated by miR-132 in DOX resistant CRC cells. Liu et al. [277] were the first and only research group to investigate how the miR-132/ERK1 axis contributes to DOX resistance in CRC cells [277]. MiR-132 expression, which is typically decreased in CRC, was significantly lower in DOX resistant CRC cell lines, whereas ERK1 mRNA and protein expression levels were significantly higher. Upon miR-132 transfection in the drug resistant cells, a significant reduction in ERK1 expression was detected, together with enhanced cell death by apoptosis, reduced cell proliferation and increased DOX sensitivity in the resistant cells. Thus, they proposed that the miR-132/ERK1 axis is responsible for respectively promoting and reducing DOX resistance in CRC [277].

In the study by Ding et al. [278], it was proposed that upregulation of miR-223 promotes DOX resistance in CRC cells via regulation of EMT, by targeting a tumor suppressor F-box and WD repeat domain containing 7 (FBXW7), as evidenced by downregulation of the epithelial marker E-cadherin and upregulation of the mesenchymal marker Vimentin. The data collected showed that the expression of miR-223 was negatively correlated with FBXW7 expression in CRC cells and tissues, and that overexpression of miR-223 reduced both the expression of FBXW7 and the sensitivity of the cells to DOX significantly [278]. In contrast, suppression of miR-223 increased the FBXW7 expression and DOX sensitivity. Thus, it was proposed that miR-223 promotes DOX resistance in CRC cells by targeting FBXW7 [278]. 

In another study, Li et al. were the first to investigate the expression of the lncRNA SLC25A25-AS1 in CRC patients with DOX or 5-FU resistance as previously discussed [204]. SLC25A25-AS1 was significantly downregulated in CRC tumour tissues, but its overexpression resulted in cell proliferation inhibition and increased DOX sensitivity, while its downregulation enhanced chemoresistance and promoted EMT by activating the ERK/MAPK signalling pathway through ERK and p38. It was established that SLC25A25-AS1 expression in both tissues and sera of CRC patients can influence DOX chemoresistance by controlling EMT changes through the ERK/MAPK signalling pathway, in both CRC tissues and cell lines. Despite it being the only published study on SLC25A25-AS1 in CRC patients resistant to DOX or 5-FU, this lncRNA may also represent a potential therapeutic target in CRC [204].

##### Apoptosis-Related Pathway

Studies have shown that miRNAs can regulate members of the BCL-2 family, which are responsible for controlling cell death and in turn contribute to drug resistance in CRC [147]. In fact, in the study by Qu et al. [279], the relationship between miRNAs and DOX resistance in CRC cell lines was investigated. From all the miRNAs found to be involved in DOX resistance, it was revealed that miRNA-195 regulates one of the anti-apoptotic regulators, Bcl-2-like protein 2 (BCL2L2). This miRNA together with others that were not further investigated were downregulated in the DOX resistant CRC cell lines. Furthermore, DOX-induced cytotoxicity was inhibited upon blockage of miRNA-195. Thus, the investigation focused on the targets of miR-195 involved in giving rise to resistance. The results showed that BCL2L2 was directly targeted by this miRNA [279], with BCL2L2 expression increasing upon inhibition of miR-195 and vice versa. DOX sensitivity increased in resistant cells having overexpression of miR-195 mimics, as cell growth decreased, and apoptotic cell death increased. Furthermore, in clinical DOX-resistant and sensitive colon cancer tissues, the expression level of miR-195 and BCL2L2 was also inversely correlated, respectively. In conclusion, despite it being the only investigation carried out both in vitro and in vivo, the data presented in this research suggested that miR-195 can regulate DOX chemosensitivity by controlling the anti-apoptosis activity via BCL2L2 [279], which in turn provide a strong rationale for the development of miRNA-based therapeutic strategies aiming to overcome CRC DOX resistance.

In another study, involving both miRNAs and lncRNAs, Zhu et al. investigated how lncRNA X-inactive specific transcript (XIST) participates in DOX resistance in CRC cells [280]. There results showed that XIST is upregulated in DOX resistant CRC cells and upon silencing this lncRNA, resistance was reversed, and apoptosis significantly increased. miR-124 was also dysregulated (downregulated) in the resistant cells and upon further analysis, they noted that XIST has two potential binding sequences of miR-124, which enabled crosstalk between the two [280]. XIST overexpression inhibited miR-124 expression, however this was reversed upon XIST knockdown. In addition, this miRNA regulated the serum and glucocorticoid-inducible kinase 1 (SGK1) protein responsible for various cellular processes [284]. SGK1 expression was positively regulated by XIST, which indicated that XIST acted as an miR-124 sponge and increased the expression of SGK1, revealing that the XIST/miR-124/SGK1 axis is responsible for modulating DOX resistance in CRC cells [280]. Despite this being the only published data on both in vitro and in vivo conditions, XIST might be a potential therapeutic target for improving the efficacy of DOX-based chemotherapy in CRC patients. In the study carried out by Xiong et al. (2021), they investigated the role of the lncRNA DANCR in promoting DOX resistance in CRC cells. Similar to what was reported by Shi et al. [269], DANCR was overexpressed in CRC tissues and cell lines, and in resistant cells, but in cells treated with DOX instead of cisplatin as the study of Shi et al. [269]. When DANCR was silenced DOX induced apoptosis increased, which resulted in decreased cell numbers in G0/G1 phase but elevated cell numbers in S and G2/M phases, while when DANCR was overexpressed, the growth of resistant cells was promoted [281]. Furthermore, overexpression of DANCR in the DOX treated cells decreased the expression of cleaved PARP and caspase 7/3, while when DANCR was silenced, apoptosis increased, and the three apoptotic proteins increased in expression. It was also demonstrated that DANCR mediated apoptosis by modulating MALAT1, with the expression of MALAT1 being downregulated upon DANCR knockdown in both cell lines and xenograft tumour tissues. Additionally, the expression of cleaved PARP and cleaved caspase 3 were downregulated in cells having overexpressed MALAT1, while the opposite was detected when MALAT1 was silenced [281]. Even though the data showed that DANCR repressed apoptosis by enhancing the expression of MALAT1, how this arose was still an open question. Further investigation showed that DANCR, as well as MALAT1, harboured multiple binding sites of the RNA-binding protein Quaking (QK), with the data showing an interaction between QK and the respective lncRNA. Eventually, it was demonstrated that QK mediated the function of DANCR on regulating MALAT1 expression and apoptotic suppression in the DOX resistance CRC cell lines. Altogether, this study identified the DOX regulated lncRNA DANCR and explored the suppressive function of DANCR on apoptosis via the QK/MALAT1 axis. However, more evidence is required to get a better insight into the mechanism of apoptosis regulation by the DANCR/QK/MALAT1 axis, as this is the only reported study in CRC [281].

##### Hippo Signaling Pathway

In the study by He et al. [282], the focus was on understanding the function of miR-135b in CRC cells with respect to DOX resistance and apoptosis so as to further push miRNA-based therapeutics closer to clinical usage. He et al. [282] showed that miR-135b could regulate CRC cell proliferation, apoptosis and DOX chemoresistance through negatively regulating Large tumor suppressor kinase 2 (LATS2) expression, a novel pro-apoptotic protein functioning through the Hippo signaling pathway. Their results were validated both on human CRC tissue samples and xenograft tumour models. They demonstrated that the upregulated expression of miR-135b represses LATS2 levels, leading to increased proliferation and DOX chemoresistance. Results from both human CRC tissue samples and xenograft tumour models showed that knockdown of endogenous LATS2 mimic the result of miR-135b upregulation to attenuate DOX-induced apoptosis. Despite these results, He et al. state that the function of miR-135b in regulating the Hippo signaling pathway via LATS2 in CRC needs to be further explored [282].

##### Other Chemoresistance Related miRNAs or lncRNAs

In the previously discussed research study by Qu et al. were the role of miR-195 and BCL2L2 in contributing to DOX resistance was investigated, data was also collected on the relationship of other miRNAs that were dysregulated in DOX resistant CRC cell lines [279]. The data showed that apart from miR-195, another three miRNAs were downregulated, miR-137, miR-127, miR-22, while miR-21, miR-592 were upregulated in DOX resistant cell lines. However, despite these results, these five miRNAs were not further investigated to determine what could have caused such dysregulation in the resistant cells.

Another study has shown that the lncRNA RAMS11 can promote resistance to topoisomerase inhibitors (in this case DOX) in CRC cell lines [283]. In DOX resistant CRC cell lines, RAMS11 expression was significantly upregulated. Due to DOX being one of the available cytotoxic drugs which target the TOP2α subunit of type IIA topoisomerases [16], Silva-Fisher et al. further investigated how overexpression of RAMS11 promoted resistance in the CRC cell lines [283], determining that RAMS11 overexpression increased TOP2α protein expression in CRC cell lines, which lead to cell lines becoming resistant to not only DOX but other chemotherapeutic drugs which also target TOP2α in their mode of action [283]. However, further work needs to be carried out to better understand the underlying mechanisms, as this is the only study in the literature which investigated how RAMS11 contributes to DOX resistance in CRC in cell lines and xenografts models [283].

## 4. Conclusions

Chemotherapy remains a challenge preventing better recovery rates for CRC patients being treated. Chemoresistance has attracted the attention of many research groups due to its clinical implications, which lead to the discovery of different molecular mechanisms responsible for chemotherapy resistance. As discusses in this review, CRC chemoresistance can arise due to overexpression of ABC transporters, different signalling pathways and cell death pathways, among others (Figure 2). It can be said that ncRNAs, particularly, miRNAs, lncRNAs and circRNAs, do have an apparent impact on modulating CRC chemoresistance, merely based on the body of evidence described above and in various other research investigations. Although not all ncRNAs associated with chemoresistance have been included in this review, since most of the focus was on recently identified ncRNAs which have not been tackled in other reviews on similar literature, there is an accumulation of evidence which implicates these RNAs in response to the different chemotherapeutics drugs discussed. The mechanisms underlying the roles of these three ncRNA families in CRC chemoresistance can be said to be fairly complex. By further exploring functional miRNAs, lncRNAs and circRNAs in CRC, new diagnostic and prognostic biomarkers will continue to be discovered. Studies should also focus on gaining a better understanding on whether 5-FU, OXA, Cisplatin and DOX resistance is arising due to specific ncRNAs which are common in various cancers, or due to different ncRNAs which target the same specific pathway/protein/ncRNA in different cancers. By identifying the different ncRNAs and their upstream or downstream mediators, further work can be carried out which focuses on specifically targeting the dysregulated endogenous miRNAs, lncRNAs and circRNAs, so as to sensitise the cancer cells to the different chemotherapeutics. Global research efforts to detect and validate novel miRNA, lncRNA and circRNA biomarkers for CRC chemoresistance should not be underestimated.

## Figures and Tables

**Table 1:** 5-FU resistance arising in CRC due to the different ncRNAs.

Mechanism	Type of ncRNA	Reported ncRNA	Alteration in CRC Cells	Validated ncRNA Targets	References
**ABC Transporter Family**	miRNA	miR-21	Upregulated	PDCD4/ABCC5/CD44	[195,196]
	miRNA	miR-361	Downregulated	FOXM1, ABCC5/10	[197,198]
	lncRNA	PVT1	Upregulated	MRP1, P-gp	[199]
	circRNA	hsa_circ_0007031	Upregulated	miR-133b/ABCC5	[200,201]
**PI3K/AKT signaling pathway**	miRNA	miR-587	Upregulated	PPP2R1B/pAKT/XIAP	[202]
	miRNA	miR-204	Downregulated	HMGA2	[203]
	circRNA	hsa_circ_0007031, hsa_circ_0007006, hsa_circ_0000504	Upregulated	AKT3	[201]
**MAPK/ERK Pathways and EMT**	lncRNA	SLC25A25-AS1	Downregulated	ERK, p38	[204]
**DNA repair mechanisms**	miRNA	miR-21	Upregulated	hMSH2	[205,206]
**Apoptosis**	miRNA	miR-361	Downregulated	Caspase 3/7	[197]
	miRNA	miR-23a	Upregulated	APAF-1	[207]
	miRNA	miR-20a	Upregulated	BNIP2	[208]
	miRNA	miR-9-5p	Downregulated	HMGA2	[209]
	lncRNA	PVT1	Upregulated	mTOR, BCL-2	[198]
	lncRNA	UCA1	Upregulated	miR-204-5p	[210]
	lncRNA	UCA1	Upregulated	miR-23b-3p/ZNF281	[211]
	miRNA	miR-20a	Upregulated	PDCD4	[212]
	lncRNA	HAND2-AS1	Downregulated	miR-20a/PDCD4	[212]
	lncRNA	DLGAP1-AS1	Upregulated	miR-149-5p/TGFB2	[213]
	circRNA	hsa_circ_0007031, hsa_circ_0000504	Upregulated	BCL2	[201]
	circRNA	circDDX17	Downregulated	miR-31-5p/KANK1	[214]
**Autophagy**	miRNA	miR-23b-3p	Downregulated	UCA1	[211]
	miRNA	miR-125b	Upregulated	CXCL12/CXCR4	[215]
	miRNA	miR-125b	Upregulated	CXCL12/CXCR4	[215]
	miRNA	miR-22	Downregulated	PARP/ATG5	[216]
	miRNA	miR-22	Downregulated	BTG1	[216]
	miRNA	miR-34a	Upregulated	HMGB1/ATG9A/ATG4B	[217]
	lncRNA	NEAT1	Upregulated	miR-34a/HMGB1/ATG9A/ATG4B	[217]
	lncRNA	SNHG6	Upregulated	miR-26a-5p/ULK1	[218]
**Hippo signaling pathway**	miRNA	miR-375-3p	Downregulated	YAP1, SP1	[219]
**Wnt/β-catenin signaling pathway**	miRNA	miR-149	Downregulated	FOXM1	[220]
	miRNA	miR-320	Downregulated	FOXM1	[221]
	miRNA	miR-375	Downregulated	FOXM1	[222]
	circRNA	circ-PRKDC	Upregulated	miR-375/FOXM1	[222]
**JAK/STAT signalling Pathway**	circRNA	hsa_circ_0000504	Upregulated	hsa-miR-485-5p/STAT3	[201]
**Other chemoresistance related ncRNAs**	miRNA	miR-375-3p	Downregulated	TYMS	[223]
	lncRNA	NEAT1	Upregulated	miR-150-5p/CPSF4	[224]
	lncRNA	NEAT1	Upregulated	H3K27ac	[225]
	lncRNA	GIHCG	Upregulated	/	[226]
	lncRNA	TUG1	Upregulated	miR-197-3p/TYMS	[227]
	lncRNA	PCAT-1	Upregulated	/	[228]
	circRNA	hsa_circ_0048234	Downregulated	miR-671-5p	[201]
	circRNA	hsa_circ_0007031	Upregulated	miR-885-3p	[201]
	circRNA	hsa_circ_0007006, hsa_circ_0000504	Upregulated	/	[201]
	circRNA	has_circ_0008509, has_circ_0084021, has_circ_0087862	Downregulated	/	[201]
	circRNA	has_circ_0008509, has_circ_0084021, has_circ_0087862	Downregulated	/	[201]
	circRNA	has_circRNA_103306	Upregulated	/	[229,230]
	circRNA	has_circRNA_406937	Downregulated	/	[230]
	circRNA	circ_0032833	Upregulated	miR-125-5p/MSI1	[231]

Abbreviations: PDCD4/10: Programmed cell death protein 4 or 10; CD44: Cluster of Differentiation 44; FOXM1: Forkhead box protein M1; ABCC5/10: ATP-Binding Cassette Subfamily C Member 5 or 10; PI3K: Phosphoinositide-3-kinase; PPP2R1B: Serine/threonine-protein phosphatase 2A subunit beta isoform; HMGA2: High mobility group protein A2; hMSH2: MutS homolog 2; APAF-1: Apoptosis-Activating Factor-1; BNIP2: BCL2 interacting protein 2; CXCL12: C-X-C Motif Chemokine 12; CXCR4: C-X-C Motif Chemokine Receptor 4; BTG1: B-cell translocation gene 1; HMGB1: High mobility group box 1; ATG9A: Autophagy Related 9A; ATG4B: Autophagy Related 4B; PARP: Poly (ADP-ribose) polymerase; ATG5: Autophagy-related gene 5; XIAP: X-linked Inhibitor of Apoptosis; YAP1: Yes-Associated Protein 1; TYMS: Thymidylate Synthase; MRP1: Multidrug resistance-associated protein 1; P-gp: P-glycoprotein 1; UCA1: Urothelial carcinoma associated 1; ZNF281: Zinc finger protein 281; TGFB2: Transforming Growth Factor Beta 2; ERK: Extracellular Signal-Regulated Kinase; H3K27ac: Histone 3 lysine 27 acetylation; TUG1: Taurine Upregulated Gene 1; PCAT-1: Prostate cancer-associated ncRNA transcript 1; SLC25A25-AS1: SLC25A25 Antisense RNA 1; NEAT1: Nuclear Enriched Abundant Transcript 1; CPSF4: Cleavage and Polyadenylation Specific Factor 4; PVT1: Plasmacytoma Variant Translocation; HAND2-AS1: HAND2 Antisense RNA 1; DLGAP1-AS1: DLGAP1 antisense 1; MSI1: Musashi1; STAT3: Signal transducer and activator of transcription 3; KANK1: Kidney ankyrin repeat-containing protein 1; AKT3: AKT Serine/Threonine Kinase 3.

**Table 2:** OXA resistance arising in CRC due to the different ncRNAs.

Mechanism	Type of ncRNA	Reported ncRNA	Alteration in CRC Cells	Validated ncRNA Targets	References
**ABC Transporter Family**	lncRNA	CACS15	Upregulated	miR-145/ABCC1	[238]
**PI3K/AKT signaling pathway**	lncRNA	LINC00152	Upregulated	miR-193a-3p/ERBB4/AKT	[239]
	circRNA	circCCDC66	Upregulated	DHX9	[240]
**Apoptosis**	miRNA	miR-20a	Upregulated	BNIP2	[208]
	miRNA	miR-153	Upregulated	FOXO3a	[241]
	miRNA	miR-425-5p	Upregulated	PDCD10	[242]
	miRNA	miR-135b	Upregulated	FOXO1	[243]
	lncRNA	BLACAT1	Upregulated	miR-519d-3p/ CREB1	[244]
	lncRNA	MEG3	Downregulated	miR-141/PDCD4	[245,246]
** Autophagy **	miRNA	miR-409-3p	Downregulated	Beclin 1	[247]
	circRNA	circHIPK3	Upregulated	miR-637/STAT3/BCL-2/Beclin1	[248]
**Wnt/β-catenin signaling pathway**	miRNA	miR-320	Downregulated	FOXM1	[221]
	lncRNA	H19	Upregulated	miR-141	[249]
** TNF-α signalling pathway **	circRNA	hsa_circ_0079662	Upregulated	hsa-mir-324-5p/HOXA6	[250]
**Glycolysis**	circRNA	hsa_circ_0005963	Upregulated	miR-122/PKM2	[251]
**Other chemoresistance related ncRNA**	miRNA	miR-203	Upregulated	ATM	[252]
	miRNA	miR-483-3p	Downregulated	FAM171B	[253]
	miRNA	miR-492	Downregulated	CD147	[254]
	miRNA	miR-200b-3p	Downregulated	TUBB3	[255]
	lncRNA	GIHCG	Upregulated	/	[226]
	lncRNA	LINC00460	Upregulated	miR-149-5p/miR-150-5p/ Mut p53	[256]
	lncRNA	CBR3-AS1	Upregulated	miR-145-5p	[257]
	lncRNA	MALAT1	Upregulated	miR-324-3p/ADAM17	[258]

Abbreviations: STAT3: Signal transducer and activator of transcription 3; HOXA6: Homeobox protein Hox-A6; DHX9: DExH-Box Helicase 9; FOXM1: Forkhead box protein M1; ABCC1: ATP-Binding Cassette Subfamily C Member 1; CACS15; Cancer Susceptibility Candidate 15; ERRB4: Erb-B2 Receptor Tyrosine Kinase 4; AKT: Protein Kinase B; BNIP2: BCL2 interacting protein 2; FOXO1: Forkhead box 1; CREB1: CAMP Responsive Element Binding Protein 1; PDCD4: Programmed cell death protein 4; BCL-2: B-cell lymphoma 2; PKM2: Pyruvate kinase; ATM: Ataxia telangiectasia mutated; FAM171B; Family With Sequence Similarity 171 Member B; TUBB3: Target β-III tubulin; MALAT1: Metastasis associated lung adenocarcinoma transcript 1; ADAM17: Disintegrin and Metalloproteinase Domain-Containing Protein 17.

**Table 3:** Cisplatin resistance arising in CRC due to the different ncRNAs.

Mechanism	Type of ncRNA	Reported ncRNA	Alteration in CRC Cells	Validated ncRNA Targets	References
**ABC Transporter Family**	lncRNA	PVT1	Upregulated	MRP1, MDR1	[262]
**Apoptosis**	miRNA	miR-153	Upregulated	FOXO3a	[241]
	lncRNA	PVT1	Upregulated	BCL-2, BAX, Cleaved Caspase-3	[262]
	lncRNA	KCNQ1OT1	Upregulated	miR-497/BCL-2	[263]
**Autophagy**	lncRNA	SNHG14	Upregulated	miR-186/ATG14	[264]
**Wnt/β-catenin signaling pathway**	miRNA	miR-203a-3p	Downregulated	β-catenin, GRG5	[265]
	lncRNA	HOTAIR	Upregulated	miR-203a-3p/β-catenin/GRG5	[265]
	lncRNA	LINC00261	Downregulated	β-catenin	[266]
**EMT**	miRNA	miR-514b-3p	Downregulated	E-cadherin, CLDN-1	[267]
	miRNA	miR-514b-5p	Upregulated	E-cadherin, CLDN-1	[267]
**ROS signalling pathway**	miRNA	miR-20a	Upregulated	ASK1/JNK	[268]
**Glycolysis**	lncRNA	DANCR	Upregulated	miR-125b-5p/HK2	[269]
** Nrf2/HO-1 pathway **	lncRNA	MIR4435-2HG	Upregulated	Nrf2/HO-1	[270]

Abbreviations: MDR1: multidrug resistance 1; MRP1: Multidrug Resistance Protein 1; PVT1: Plasmacytoma Variant Translocation; FOXO3a: Forkhead box O3a; KCNQ1OT1: KCNQ1 Opposite Strand/Antisense Transcript 1; SNHG14: Small Nucleolar RNA Host Gene 14; CLDN-1: Claudin 1; HOTAIR: HOX Transcript Antisense RNA; ASK1: Apoptosis signal-regulating kinase 1; JNK: c-Jun N-terminal kinase; DANCR: Differentiation antagonising non-coding RNA; HK2: Hexokinase 2; Nrf2: Nuclear factor erythroid 2-related factor 2; HO-1: Heme oxygenase-1; MIR4435-2HG: MIR4435-2 Host Gene.

**Table 4:** DOX resistance arising in CRC due to the different ncRNAs.

Mechanism	Type of ncRNA	Reported ncRNA	Alteration in CRC Cells	Validated ncRNA Targets	References
**ABC Transporter Family**	miRNA	miR-522	Downregulated	ABCB5	[274]
	miRNA	miR-29a	Downregulated	MDR1/P-gp	[275]
**PI3K/AKT signaling pathway**	miRNA	miR-29a	Downregulated	PTEN	[275]
	miRNA	miR-223	Upregulated	FBXW7	[276]
**MAPK/ERK Pathways and EMT**	miRNA	miR-132	Downregulated	ERK1	[277]
	miRNA	miR-223	Upregulated	FBXW7	[278]
	lncRNA	SLC25A25-AS1	Downregulated	ERK, p38	[204]
**Apoptosis**	miRNA	miR-195	Downregulated	BCL2L2	[279]
	lncRNA	XIST	Upregulated	SGK1/miR-124	[280]
	lncRNA	DANCR	Upregulated	QK/MALAT1	[281]
**Hippo signaling pathway**	miRNA	miR-135b	Upregulated	LATS2	[282]
**Other chemoresistance related ncRNA**	miRNA	miR-137, miR-127, miR-22	Downregulated	/	[279]
	miRNA	miR-21, miR-592	Upregulated	/	[279]
	lncRNA	RAMS11	Upregulated	TOP2α	[283]

Abbreviations: SGK1: Serum/Glucocorticoid Regulated Kinase 1; TOP2α: Topoisomerase IIA; ERK: Extracellular Signal-Regulated Kinase; XIST: X-inactive specific transcript; DANCR: Differentiation antagonising non-coding RNA; QK: Quaking; MALAT1: Metastasis associated lung adenocarcinoma transcript 1; FBXW7: F-box and WD repeat domain containing 7; LATS2: Large tumor suppressor kinase 2; SGK1: Serum/Glucocorticoid Regulated Kinase 1; BCLCL2: B-cell lymphoma like protein 2; ERK1: Extracellular Signal-Regulated Kinase 1; PTEN: Phosphatase and tensin homolog; MDR1: multidrug resistance 1; P-gp: P-glycoprotein 1; ABCB5: ATP-binding cassette sub-family B member 5; RAMS11: RNAs Associated with Metastasis 11; SLC25A25-AS1: SLC25A25 Antisense RNA 1.
